# Distinct Subfamilies of Odorant Binding Proteins in Locust (Orthoptera, Acrididae): Molecular Evolution, Structural Variation, and Sensilla-Specific Expression

**DOI:** 10.3389/fphys.2017.00734

**Published:** 2017-09-26

**Authors:** Xingcong Jiang, Jürgen Krieger, Heinz Breer, Pablo Pregitzer

**Affiliations:** ^1^Institute of Physiology, University of Hohenheim, Stuttgart, Germany; ^2^Department of Animal Physiology, Institute of Biology/Zoology, Martin Luther University Halle-Wittenberg, Halle, Germany

**Keywords:** locust, *Schistocerca gregaria*, odorant binding protein, evolution, structure, sensilla

## Abstract

Odorant binding proteins (OBPs) play an important role in insect olfaction, facilitating transportation of odorant molecules in the sensillum lymph. While most of the researches are concentrated on Lepidopteran and Dipteran species, our knowledge about Orthopteran species is still very limited. In this study, we have investigated OBPs of the desert locust *Schistocerca gregaria*, a representative Orthopteran species. We have identified 14 transcripts from a *S. gregaria* antennal transcriptome encoding SgreOBPs, and recapitulated the phylogenetic relationship of SgreOBPs together with OBPs from three other locust species. Two conserved subfamilies of classic OBPs have been identified, named I-A and II-A, exhibiting both common and subfamily-specific amino acid motifs. Distinct evolutionary features were observed for subfamily I-A and II-A OBPs. Surface topology and interior cavity were elucidated for OBP members from the two subfamilies. Antennal topographic expression revealed distinct sensilla- and cellular- specific expression patterns for SgreOBPs from subfamily I-A and II-A. These findings give first insight into the repertoire of locust OBPs with respect to their molecular and evolutionary features as well as their expression in the antenna, which may serve as an initial step to unravel specific roles of distinct OBP subfamilies in locust olfaction.

## Introduction

In insects, the process of olfactory signal processing begins in hair-like cuticle appendages, called sensilla, located mainly on the antennae and palps (Steinbrecht, 1996; Hansson and Stensmyr, 2011; Suh et al., 2014). Olfactory sensory neurons (OSNs) project their dendrites into the lumen of the sensillar hairs, which is filled with sensillum lymph (Hansson and Stensmyr, 2011; Suh et al., 2014). The hydrophobic odorant molecules enter the sensillum via the porous cuticle and have to pass the aqueous lymph till reaching the chemosensory membrane of the sensory neurons (Vogt et al., 1999; Leal, 2013; Suh et al., 2014). This passage is thought to be mediated by small soluble proteins enriched in the sensilla lymph, the so called odorant binding proteins (OBPs), which are produced and secreted by accessory cells (Pelosi et al., 2006, 2017). OBPs are polypeptides comprised of ~110–200 amino acids; usually they exhibit a considerable degree of sequence divergence. Based on the number of conserved cysteine (C)-residues, several subtypes are discriminated. Whereas, the pattern of six conserved C-residues represents a hallmark of classic OBPs (Pelosi et al., 2006), OBPs with more or with less C-residues are designated as plus-C and minus-C OBPs (Zhou et al., 2004; Foret and Maleszka, 2006). In addition, atypical OBPs have been classified which may originate from a fusion of two classic OBPs (Xu et al., 2003; Vieira and Rozas, 2011). Typically, the tertiary structure of insect OBPs consists of six α-helices forming an interior binding cavity. This structure is maintained and stabilized by disulfide bridges formed by conserved C-residues (Leal et al., 1999; Scaloni et al., 1999; Sandler et al., 2000). However, OBP structures with more than six helices have been reported (Horst et al., 2001; Lagarde et al., 2011). It is also proposed that the C-terminal domain that is variable in length can spatially interfere with the interior binding cavity and thus may affect the ligand binding mechanism (Damberger et al., 2000; Horst et al., 2001; Tegoni et al., 2004; Pelosi et al., 2017).

Most of our current knowledge of insect OBPs is based on studies of species from the taxa Lepidoptera and Diptera (Hekmat-Scafe et al., 2002; Vogt et al., 2002; Leal, 2013; Pelosi et al., 2017). The desert locust, *Schistocerca gregaria* is a representative of the taxa Orthoptera, which is quite distant from the orders Lepidoptera and Diptera on the phylogenetic scale (Wheeler et al., 2001; Vogt et al., 2015) and as hemimetabolous insects their developmental process differ significantly from that of holometabolous insects. Very little is known about OBPs in Orthoptera; only a limited number of sequences have recently been reported for a few locust species: *Locusta migratoria* (Ban et al., 2003; Xu et al., 2009; Yu et al., 2009), *Oedaleus asiaticus* (Zhang et al., 2015), and *Ceracris kiangsu*. Information about the expression of locust OBPs in the olfactory sensilla is limited to LmigOBP1, which was found to be expressed in sensilla trichodea and sensilla basiconica (Jin et al., 2005). Concerning another olfactory sensillum type, the sensilla coeloconica, a possible expression of OBPs has rarely been documented even in holometabolous insect species (Larter et al., 2016). Incidentally, the crystal structure of locust OBPs has only been resolved for LmigOBP1, which establishes a unique seven-α-helices structure (Zheng et al., 2015). The possibility of structural differences between locust OBPs is still an open question.

In order to extend our knowledge about OBPs in Orthopteran locust species, in the current study we have performed a systematic characterization of locust OBPs with respect to the molecular evolution, structural variation and sensilla-specific expression. Based on the OBP sequences of *S. gregaria* newly identified from an antennal transcriptome and the documented OBP sequences from other locust species, we conducted a phylogenetic analysis of the current locust OBP repertoire. The emerging two subfamilies of classic OBPs were compared for sequence divergence, selection pressure and variation of the predicted tertiary structure in detail. Analysis of the topographic expression pattern revealed that the molecular and phylogenetic distinctness between the two subfamilies are accompanied by a sensilla-specific expression pattern.

## Materials and methods

### Identification of *S. gregaria* OBP transcripts

A *S. gregaria* antennal transcriptome database was generated comprising a total of 55,060 contigs with an N50 of 2,223 bp. The strategy of homology-mining was adopted to identify the candidate OBP transcripts. We retrieved documented OBPs from different insect species including *Anopheles gambiae* (AgamOBPs, Diptera), *Apis mellifera* (AmelOBPs, Hemiptera), *Drosophila melanogaster* (DmelOBPs, Diptera), *Tribolium castaneum* (TcasOBPs, Coleoptera), *Acyrthosyphon pisum* (ApisOBPs, Hemiptera), *Bombyx mori* (BmorOBPs, Lepidoptera) (Vieira and Rozas, 2011), *Blattella germanica* (BgerOBPs, Blattaria) (Niu et al., 2016), and *Zootermopsis nevadensis* (ZnevOBPs, Isoptera) (Terrapon et al., 2014), as well as from three other locust species, including *L. migratoria* (LmigOBPs) (Ban et al., 2003; Yu et al., 2009), *O. asiaticus* (OasiOBPs) (Zhang et al., 2015), and *C. kiangsu* (CkiaOBPs). Using the collected sequences as queries, we conducted a local tBLASTx search on BioEdit 7.2.5 against the transcriptome database with an *E*-value < 10^−5^. Annotation of the screened contigs was inspected by performing tBLASTx and BLASTp search against non-redundant (nr) protein database in NCBI (Bethesda, MD, USA). The extracted contigs which putatively encode OBPs were in turn used as new queries to identify additional candidates using tBLASTx and BLASTp methods. Open reading frames in the identified OBP transcripts were inspected by Genamics Expression (Hamilton, New Zealand). Accession numbers for the newly identified SgreOBPs and other locust OBPs are deposited in the Supplementary Material.

### Characterization of consensus amino acid motifs

Signatures of sequence divergence underlying locust subfamily I-A and II-A OBPs were addressed by identifying consensus amino acid motifs. Toward that goal, the online MEME SUITE v. 4.11.2 (http://meme-suite.org/tools/meme) was used (Bailey et al., 2009), with the default setting (motif width: 6–50 amino acids; motif distribution: zero or one occurrence per sequence). The output comprised six consensus motifs which was ascertained to be sufficient to recapitulate the sequence information of subfamily I-A and II-A. The identified six motifs were also utilized to target sequences of the locust OBP repertoire to obtain the motif match degree (match *E*-value) using MAST module (Motif Alignment and Search Tool) implemented in MEME SUITE. The motif match *E*-value assesses statistical significance of the consensus motif toward a targeted sequence based on its log likelihood level and the occurrence frequencies of background amino acids. The default statistical significant threshold setting was e^−5^.

### Phylogenetic analysis

The OBP amino acid sequences from four hitherto documented locust species were utilized to recapitulate the phylogenetic relationship: 16 from *L. migratoria*, 15 from *O. asiaticus*, 7 from *C. kiangsu* and the currently identified 14 candidates from *S. gregaria*. Amino acid sequences of OBPs from the four locust species are deposited in the Supplementary Material. The predicted signal peptide (SP) on the N-terminal domain was deleted before the sequences being further investigated due to two reasons: (1) SP is cut off in post-translational modification when the protein is secreted into the sensillum lymph; (2) SP exhibits a certain degree of sequence divergence but may contain limited bio-information (Vieira et al., 2007). Prediction of SP was based on SignalP 4.1 (http://www.cbs.dtu.dk/services/SignalP/) (Petersen et al., 2011). Multiple sequence alignments were conducted by MAFFT v. 7 (http://mafft.cbrc.jp/alignment/server/) using the algorithm E-INS-I, which is accuracy favored and is suitable for sequences with multiple conserved domains (Katoh and Standley, 2013). After the alignment, Gblocks v. 0.91b (http://molevol.cmima.csic.es/castresana/Gblocks_server.html) was used to inspect the poorly aligned sites and divergent regions (Castresana, 2000). To search an optimal amino acid substitution model, we chose the Find Best Protein Model implemented in MEGA 6.0 which performs a comprehensive parametric assessment (e.g., BIC scores, AICc value, lnL value) (Tamura et al., 2013). The Whelan and Goldman model (WAF), discrete GAMMA distribution (G) and an assumed fraction of evolutionary invariable sites (I) was considered to describe the substitution best. RAxML v. 8.2.9 implemented in the CIPRES Science Gateway (https://www.phylo.org/) was used for the locust OBP phylogeny construction (Miller et al., 2012; Stamatakis, 2014). A search of best scoring maximum likelihood tree (-f a) was launched, supported by 1,000 rapid bootstrap iterations (autoMRE based bootstopping criterion). The generated maximum likelihood tree was graphically edited by FigTree v. 1.4.3 (http://tree.bio.ed.ac.uk/software/figtree/). A similar strategy was applied to analyze the phylogenetic relationship between locust OBPs and OBPs from eight other insect species. In brief, SignalP, MAFFT, and Gblocks were used to prepare the multiple sequence alignment; RAxML was responsible for building the maximum likelihood tree (-f a, 1,000 iteration) using the proposed best fitting substitution model (WAG+G+I) by MEGA.

### Selection constraint on locust OBP repertoire

The nucleotide coding sequences underlying the locust OBP repertoire (see Supplementary Material) were aligned in accordance with the multiple sequence alignment from the above mentioned phylogenetic analysis using TranslatorX (http://translatorx.co.uk/). The sequence order of alignment was guided by the constructed phylogenetic tree mentioned above. The signatures of selection regime acting on sequences of the locust OBP phylogeny were estimated by resolving three principle concepts: the non-synonymous substitution rate (dN), synonymous substitution rate (dS) and the ω rate (dN/dS). Toward that, HyPhy batch program was utilized which implements maximum likelihood estimate and post-likelihood ratio test (Kosakovsky Pond et al., 2005). A local fit model (MG94xREV_3x4 substitution model) was adopted (Kosakovsky Pond et al., 2009), and each single branch in the locust OBP phylogeny was assigned with a unique set of dN and dS values, assuming the branch-to-branch variant ω rates. To support the local fit model, we additionally conducted a coarse estimate of the ω rate using the alternative global fit model, assuming invariable ω rate shared by different phylogenetic branches. A likelihood ratio test compared the results obtained from two distinct models, and strongly favored the local fit model (*P* = 10^−3^). Normality distribution of dN, dS, and the ω rates was assessed by D'Agostino-Pearson test, and the statistical difference was evaluated by non-parametric Mann-Whitney *U*-test. GraphPad Prism 5.0 was used to analyze the data and generate the diagrams (San Diego, CA, USA).

### Synthesis of riboprobes for *in situ* hybridization

The coding sequences of six SgreOBPs from locust OBP subfamily I-A and II-A were amplified, sequenced and then cloned into the pGEM-T vectors (Invitrogen) for subsequent transcription. Linearized pGEM-T vectors carrying SgreOBPs coding sequences were utilized to synthesize digoxigenin (Dig) and biotin (Bio) labeled anti-sense and sense RNA probes using the T7/SP6 RNA transcription system (Roche, Germany). The sense (s) and antisense (as) primers used for amplication of the SgreOBP sequences were:

SgreOBP1 s, ctgggacgtcaacatgaaact;
SgreOBP1 as, aatgcacgaactaccaggctg;
SgreOBP5 s, ggccgcgccgtcttctcataagga;
SgreOBP5 as, cggccctggcgcagcacctgcatt;
SgreOBP6 s, acagcacaccaccgtcacac;
SgreOBP6 as, ggtgcttgcttgaagaggcac;
SgreOBP10 s, gcgtatcacccggctgtgta;
SgreOBP10 as, agtctcacctctgccagcga;
SgreOBP11 s, tggaccgcacgacaacaaca;
SgreOBP11 as, cgatagcgtatgccctttcac;
SgreOBP14 s, ctgttgggtgcagtcctgtt;
SgreOBP14 as, gtcgtgacagctcctccactg

### *In situ* hybridization

Antennae of adult *S. gregaria* were dissected and embedded in Tissue-Tek O.C.T. Compound (Sakura Finetek Europe, The Netherlands). Cryosections at 12 μm were thaw mounted on SuperFrost Plus slides (Menzel-Gläser, Braunschweig, Germany) at −21°C (Jung CM300 cryostat). RNA *in situ* hybridization (ISH) was conducted as previously reported (Yang et al., 2012; Guo et al., 2013; Jiang et al., 2016). Section were fixed (4% paraformaldehyde in 0.1 M NaHCO_3_, pH 9.5) at 4°C for 22 min. The following consecutive steps were conducted at room temperature: a wash for 1 min in PBS (phosphate buffered saline = 0.85% NaCl, 1.4 mM KH_2_PO_4_, 8 mM Na_2_HPO_4_, pH 7.1), an incubation for 10 min in 0.2 M HCl, another wash for 1 min in PBS, an incubation for 10 min in acetylation solution (0.25% acetic anhydride freshly added in 0.1 M triethanolamine) and washes for three times in PBS (3 min each). Sections were prehybridized for 1 h at 60°C in hybridization buffer (50% formamide, 5 × SSC, 50 μg/ml heparin, and 0.1% Tween-20). 100 μl hybridization solution containing the labeled RNA in hybridization buffer was placed onto the tissue section. A coverslip was placed on top and slides were incubated in a moister box at 60°C overnight (18–20 h). After hybridization, slides were washed twice for 30 min in 0.1 × SSC at 60°C, then each slide was treated with 1 ml 1% blocking reagent (Roche) for 40 min at room temperature.

Visualization of Dig-labeled probe hybridizations was achieved by using an anti-Dig alkaline phosphatase (AP) conjugated antibody (1:500, Roche) and NBT/BCIP substrate. Antennal sections were analyzed on a Zeiss Axioskope2 microscope (Zeiss, Oberkochen, Germany) equipped with Axiovision software. For two-color FISH visualization of hybridized probes was performed by using an anti-Dig AP-conjugated antibody in combination with HNPP/Fast Red (Roche) for Dig-labeled probes and an anti-biotin streptavidin horse radish peroxidase-conjugate together with fluorescein-tyramides as substrate (TSA kit, Perkin Elmer, MA, USA) for Bio-labeled probes. Sections from FISH experiments were analyzed with a Zeiss LSM510 Meta laser scanning microscope (Zeiss, Oberkochen, Germany). Confocal images stacks were processed by ZEN 2009 software. The pictures shown represent projections of optical planes selected from confocal image stacks. For clear data presentation, images were only adjusted in brightness and contrast. Antennal sections of both male and female antennae were analyzed using each generated probe. No obvious difference between sexes regarding the labeling intensity and labeling pattern was observed. Thus, only the images of male antenna were adopted in this study.

### Structure modeling and electrostatic potential

*In silico* simulation of OBP tertiary structure was performed by I-TASSER server (http://zhanglab.ccmb.med.umich.edu/I-TASSER/) (Roy et al., 2010), which implements the iterative template threading refinement making full use of established homologous protein structures. PyMol was used to visualize the simulated protein tertiary structures (DeLano, 2002). The molecular surface was solvent excluded and the solvent radius was set 1.4 as default. APBS plug (Unni et al., 2011) implemented in PyMol was employed to calculate the surface electrostatic potentials in the range of −6 to 6 kT/e, and was presented as blue-red hue gradient.

## Results

### Identification, C-skeleton pattern and phylogenetic relationship of locust OBPs

**Figure 1:**
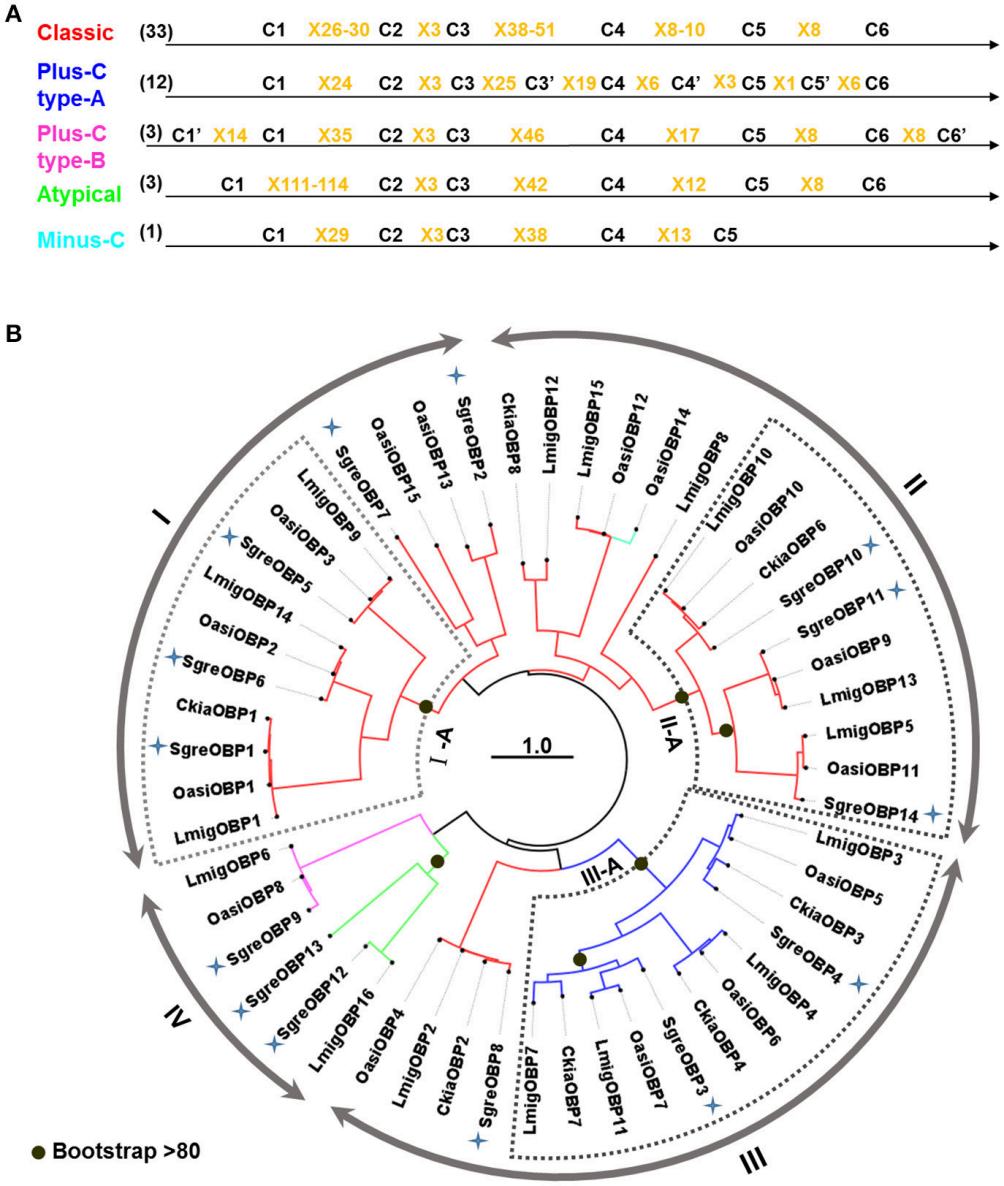
C-residue skeletons and phylogeny of OBPs from four locust species. **(A)** OBPs subtypes were categorized based on the number of conserved C-residues. C-skeleton patterns are based on the multiple sequence alignment of 52 OBP sequences from four locust species. C-residues conserved in all OBPs are shown as C1-C6 in black characters; additional C-residues conserved in the two plus-C OBP types are shown as C'; amino acid between two C-residues are shown as X plus the number of amino acid. The number of each OBP subtype is given in the parenthesis. **(B)** The phylogenetic tree was constructed using the maximum likelihood algorithm supported by 1,000 bootstrap replicates. OBP sequences utilized to generate the tree were derived from four locust species: 14 from *Schistocerca gregaria* (SgreOBPs), 16 from *Locusta migratoria* (LmigOBPs), 15 from *Oedaleus asiaticus* (OasiOBPs) and 7 from *Ceracris kiangsu* (CkiaOBPs). Four primary families (I-IV) are denoted by arrow lines. Further classification of three subfamilies (I-A, II-A, and III-A) was based on the over 80% bootstrap support at the internal node (indicated by black dots). Inner branches in different colors represent OBP subtypes in **(A)**: red, classic OBPs; blue, plus-C OBPs type-A; magenta, plus-C OBPs type-B; green, atypical OBPs; cyan, minus-C OBP. Newly identified SgreOBPs are denoted by blue crosses. The tree is midpoint rooted. Scale bar represents one amino acid substitution per site.

Toward an identification of OBPs from *S. gregaria* and a comprehensive characterization of OBPs in locust species, we have performed a homology-based data mining of an antennal transcriptome which resulted in 14 transcripts putatively encoding SgreOBPs. Subsequently, a multiple sequence alignment was conducted addressing the amino acid sequences of the newly identified SgreOBPs together with hitherto documented OBPs from three other locust species: 16 from *L. migratoria*, 15 from *O. asiaticus* and 7 from *C. kiangsu*. Several OBP subtypes could be categorized based on the number of conserved C-residues (Figure 1A). First, 33 OBPs were classified as classic OBPs comprising six conserved C-residues, the hallmark of classic OBPs. Second, 15 OBPs were categorized in two types of plus-C OBPs harboring more than six conserved C-residues. Finally, only one minus-C OBP with less than six conserved C-residue and three atypical OBPs with extraordinary long stretches between conserved C1 and C2 were identified.

As a next step, we analyzed the phylogenetic relationship of the locust OBP repertoire by constructing a phylogenetic tree utilizing the maximum likelihood algorithm and bootstrap iterations. The emerging picture indicated that the repertoire of locust OBPs can be divided into four major families (I–IV), which apparently split at the internal nodes (Figure 1B). We further classified three additional subfamilies (I-A, II-A, and III-A), based on the presence of higher bootstrap support (above 80%) on the divergent nodes. It is noteworthy that subfamily I-A and II-A both represent classic OBPs and each subfamily apparently comprise three distinct groups with 3–4 orthologous OBPs from different locust species (Figure 1B). Within each subfamily, the sequence identity between OBPs from different groups ranged from 28 to 35%; OBP members within each ortholog group exhibit generally above 80% sequence identity. Incidentally, plus-C OBPs type-A converged onto a subfamily III-A and segregated from their counterparts plus-C OBPs type-B and classic OBPs. Together, the data indicate a considerable degree of orthology in the OBP repertoires across the four analyzed locust species and no marked species-specific expansion within the OBP phylogeny.

### Elucidation of subfamily-specific consensus amino acid motifs

**Figure 2:**
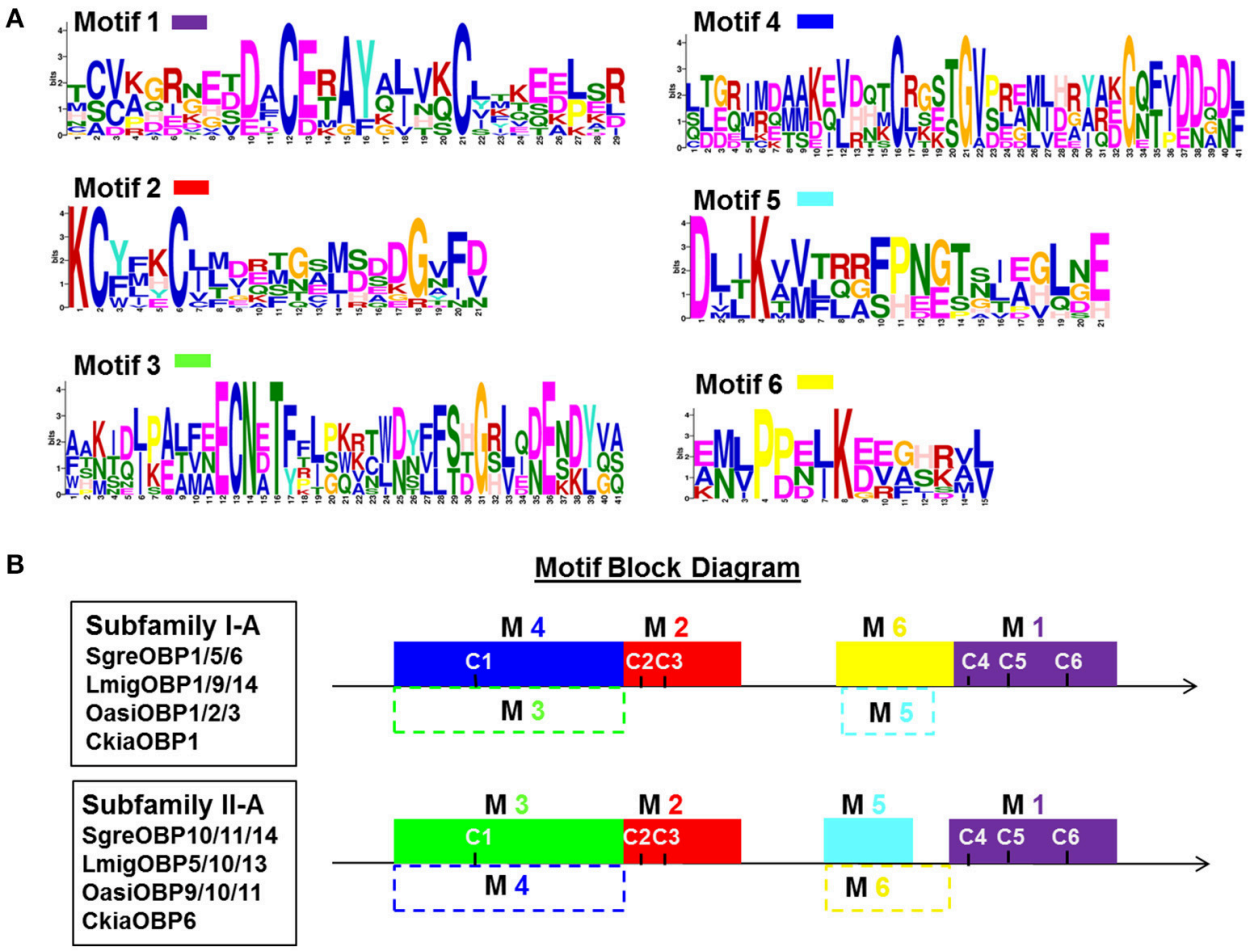
Identification and position of consensus amino acid motifs for subfamily I-A and II-A OBPs. **(A)** Six amino acid motifs with various widths were identified *de novo* to recapitulate the subfamily I-A and II-A OBP sequence signature (classification see Figure 1). The height of an amino acid character is proportional to the degree of conservation in the consensus sequences. **(B)** Position of identified consensus motifs (M1–M6) in the polypeptide chain of subfamily I-A and II-A OBPs. C1–C6 indicate the position of the conserved C-residues. Motif 1 and motif 2 adequately match the repertoire of OBP sequences in both subfamily I-A and II-A. In contrast, motifs combination M4 (blue) and M6 (yellow) specifically match subfamily I-A, whereas motifs M3 (green) and M5 (cyan) are specific for subfamily II-A OBP sequences. Dash lined blocks indicates unfitness of a particular motif to the target sequences (*E*-value above e^−5^; default statistical significant level). Obtained *E*-values for each motif are given in Figure S1.

To better elucidate the clustering regime of individual subfamilies, we analyzed the consensus amino acid motifs characteristics underlying subfamily I-A and II-A OBPs. The local consensus motifs were calculated by recapitulating repeatedly occurring sequence patterns along OBP sequences. Six consensus motifs with various widths were identified and localized at distinct positions (Figure 2). The motif 1 and motif 2 appeared as common motifs in all OBPs of both subfamilies, whereas the other four motifs specifically fit either the repertoire of subfamily I-A OBPs (motif combination 4 and 6) or the repertoire of subfamily II-A OBPs (motif combination 3 and 5). Therefore, two less divergent sequence domains were unraveled by the presence of motif combination 1 and 2, spanning the domains of C2–C3 and C4–C6. In contrast, the sequence domains close to the N-terminus (42 amino acids, motif 3 and motif 4) and ahead of C4 (11–15 amino acids, motif 5 and motif 6) appeared to be more divergent.

Utilizing the six identified consensus motifs in Figure 2 we have quantified the sequence divergence for the locust OBP repertoire at a local motif scale (Figure S1). Apart from subfamilies I-A and II-A, the common motif 1 and motif 2, especially the latter, recapitulate sequence information present in many of the other locust OBPs analyzed (*E*-value below 10^−5^) indicating particular phylogenetic conservation of these regions. Not surprisingly, the subfamily-specific motifs 3–6 failed to match OBP members (*E*-value above 10^−5^) other than subfamily I-A and II-A OBPs, despite a small number of OBPs in family I and family II (Figure S1). Taken together, the motif analysis unraveled the presence of both stabilized and diversified domains residing on the global sequences.

### Selection pressure and orthology evolution of locust subfamily I-A and II-A

**Figure 3:**
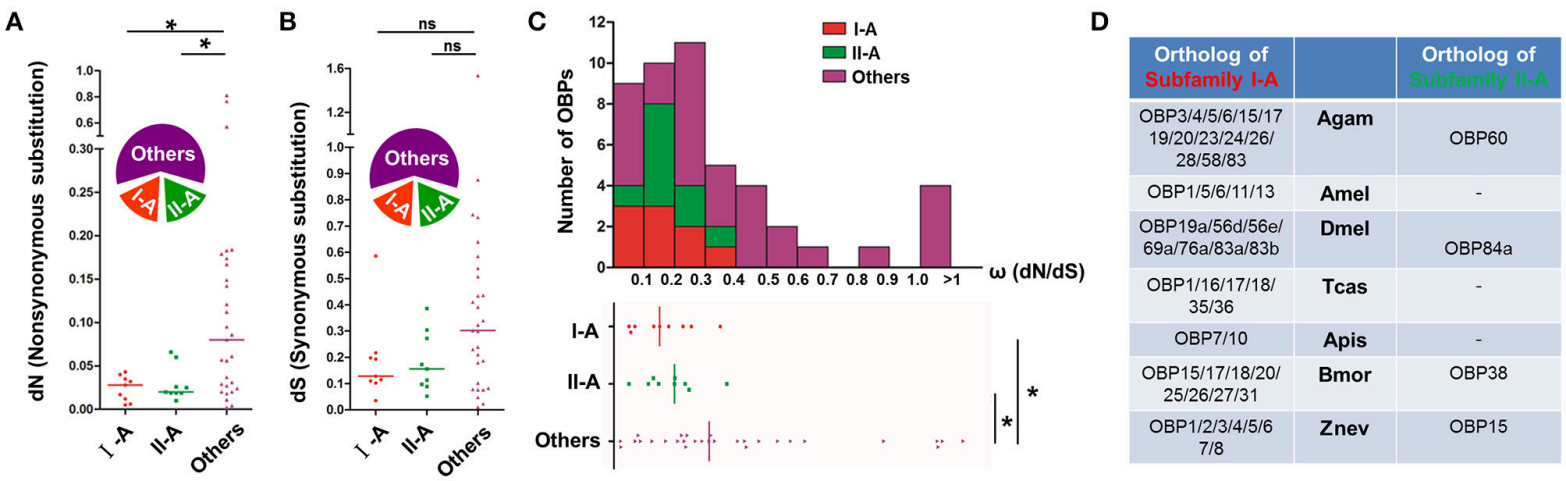
Selection constraints and orthology evolution of locust subfamily I-A and II-A. **(A,B)** Locust subfamily I-A and II-A OBPs exhibit a reduced dN rate, but a similar dS rate in comparison with the other locust OBPs. “Others” include those OBPs that do neither belong to subfamily I-A nor to subfamily II-A. The relative fraction included in each OBP group is illustrated by the wedges diagrams. Non-synonymous substitution rates (dN) and synonymous substitution rates (dS) were calculated across the locust OBP repertoire. The median level is indicated by lines. ^*^p < 0.05; ns, *p* > 0.05; two-tailed Mann-Whitney *U*-test. Detailed data of this analysis are given in Figure S2. **(C)** Proportional distribution of ω rates for the locust OBP repertoire. The majority of OBPs (~90%) fall into a ω range of 0–0.7. Yielded ω ratios (dN and/or dS ≠ 0) and the median level are displayed at the bottom. ^*^p < 0.05; two-tailed Mann-Whitney *U*-test. Nucleotide sequences utilized in this analysis are given in the supplementary material. **(D)** Orthologs of locust subfamily I-A and II-A OBPs in seven other insect species. It is noted that the complete genome has been sequenced for the seven inspected species, namely, *Anopheles gambiae* (Agam), *Apis mellifera* (Amel), *Drosophila melanogaster* (Dmel), *Tribolium castaneum* (Tcas), *Acyrthosyphon pisum* (Apis), *Bombyx mori* (Bmor), and *Zootermopsis nevadensis* (Znev). Orthology assignment was obtained by using EggNOG 4.5.1 which performed a hierarchical orthologous annotation (Huerta-Cepas et al., 2016). The criteria *E*-value for assessing orthologous relationship of locust subfamily I-A is set to e^−20^, while e^−10^ for subfamily II-A. Short bar denotes that there are no appropriate hints that could be assigned as orthologous OBPs. Nomenclature of OBPs for the seven inspected insect species conforms to Vieira and Rozas (2011) and Terrapon et al. (2014).

The appearance of two distinct conserved subfamilies in the locust OBP phylogeny, coupled with the clustering pattern of different ortholog groups is presumably a consequence of particular selection regimes. To prove this notion, we have tried to quantify the strength of selection pressure acting on genes encoding the locust OBP repertoire. We analyzed three principal concepts which reflect the selection pressure, namely, the non-synonymous substitution rates (dN), the synonymous substitution rates (dS) and the ω rates (dN /dS) (Figure 3). We found a significantly reduced median dN level for both subfamily I-A (dN = 0.030, *U* = 60, *p* = 0.016, Mann-Whiteny *U*-test) and subfamily II-A (dN = 0.028, *U* = 60, *p* = 0.016, Mann-Whiteny *U*-test), in comparison with that of other OBP members (dN = 0.085, Figure 3A). However, the median dS level appeared to be quite similar among subfamily I-A (dS = 0.12, *p* = 0.154, *U* = 88.5, Mann-Whiteny *U*-test), subfamily II-A (dS = 0.16, *U* = 86, *p* = 0.131, Mann-Whiteny *U*-test) and the other OBP members (dS = 0.31, Figure 3B). For the ω rates, the values ranged from 0 to 0.7 for nearly 90% of locust OBPs (Figure 3C), which is indicative of purifying selection acting on locust OBP repertoire in general. For a few exceptions, ω rates larger than one were found which may indicate a positive selection. Notably, median ω rates for OBPs of subfamily I-A (ω = 0.18, *U* = 63, *p* = 0.021, Mann-Whiteny *U*-test) and subfamily II-A (ω = 0.22, *U* = 69, *p* = 0.036, Mann-Whiteny *U*-test) were significantly reduced in comparison with other OBP members in the phylogeny (ω = 0.35, Figure 3C).

Exposed to a similar selection regime, we wondered if orthologous OBPs in other species would undergo similar divergent events in relation to the two locust OBP subfamilies. To address the issue, we made a phylogenetic analysis of the two locust OBP subfamilies and the reference OBPs derived from 8 other insect species which gradually emerged in the course of insect evolution. The analysis revealed that locust subfamily II-A OBPs remained on an intact clade without intermingling with reference OBP genes on the newly constructed phylogenetic tree (Figure S3). A different result was obtained for the subfamily I-A: the original clustering relationship of ortholog groups in locust phylogeny was disrupted and altered with a complex re-clustering pattern integrating reference OBPs. The orthologous relationship (Theißen, 2002) of OBPs between the two locust subfamilies and other species was also inferred. It is found that the number of locust subfamily I-A orthologous OBPs in the inspected insect species expanded considerably, and exhibited a many-to-many orthologous relationship with locust subfamily I-A (Figure 3D), with *A. pisum* as apparent exception likely due to a smaller OBP gene repertoire (Zhou et al., 2010). In contrast, the number of locust subfamily II-A orthologous OBPs in other species apparently decreased, and displayed a 1-to-many or 0-to-many orthologous relationship with locust subfamily II-A (Figure 3D). Moreover, it was found that locust subfamily II-A OBPs and their orthologous OBPs may share a common ancestor verified by the convergence of a mono phylogenetic clade with the bootstrap support above 70% at the basal divergent node (Figure S3). However, the common ancestral status for locust subfamily I-A OBPs and their orthologous OBPs appeared ambiguous because of the absence of evident bootstrap support (Figure S3). In sum, our results provide evidence that locust subfamily I-A and II-A OBPs are subject to mutually similar strengthened purifying selection, whereas distinct divergent events occur during evolution of their orthologous OBPs in other species.

### Prediction of tertiary structures for OBPs in subfamily I-A and II-A

The intriguing sequence and evolutionary characteristics underlying locust subfamily I-A and II-A OBPs inspired us to explore the possible concurrent variation of their tertiary structures. Therefore, we have simulated the tertiary structures for OBP members from both two subfamilies covering different ortholog groups and locust species. Parametric estimates toward the accuracy and reliability of the structure prediction was scrutinized, which permitted to investigate structural variation as an exploratory trial. To unravel structural variation between the two subfamilies, we superimposed the backbone structures of those simulated OBPs to LmigOBP1, the hitherto only established crystal structure for the locust OBP repertoire (Zheng et al., 2015). The averaged RMSD score obtained by imposing subfamily II-A OBPs to LmigOBP1 (2.8) doubled that of imposing subfamily I-A OBPs to LmigOBP1 (1.39 in average, Figure S4), indicating an enhanced structural similarity within one subfamily.

**Figure 4:**
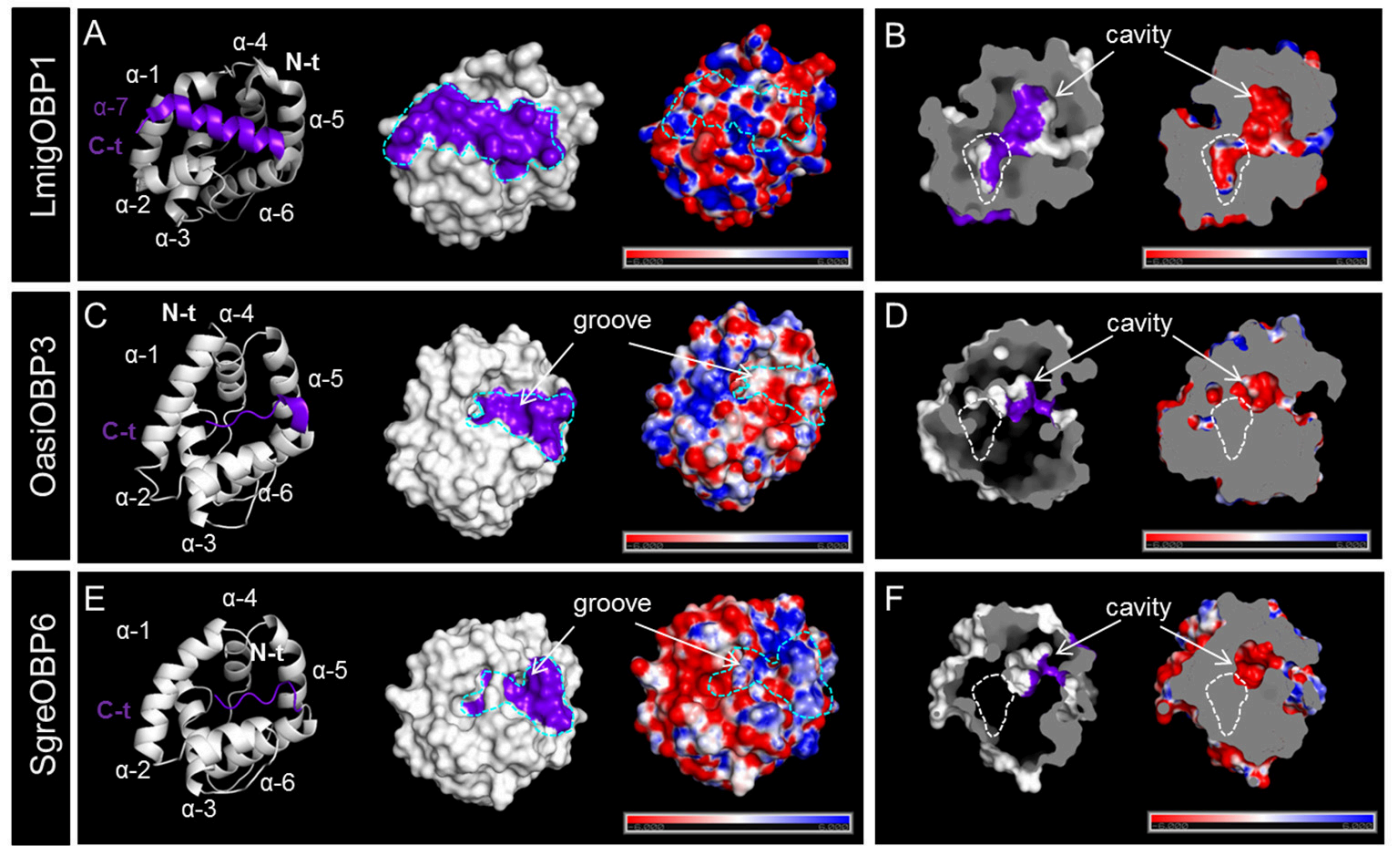
Variations in the C-terminal domain and the interior cavity of subfamily I-A OBPs. **(A,C,E)** Comparison of the backbone structures, surface topologies and surface potentials of LmigOBP1, OasiOBP3 and SgreOBP6 which represent the three different ortholog groups in subfamily I-A. The C-terminal domains (see also Figure S4A) are highlighted in purple on both the backbone structures (left) and the molecular surfaces (middle). The dash line in cyan sketches the surface topology of the C-terminal domain (middle and right). Left and middle: an additional α-helix (α-7) is formed by the prolonged C-terminus in LmigOBP1 (Zheng et al., 2015) **(A)**. Instead of a seventh α-helix, the shortened C-terminus in OasiOBP3 **(C)** and SgreOBP6 **(E)** are likely to constitute a groove structure on the collapsed surface. Right: a map of electrostatic potential on the molecular surface. The electrostatic potential pattern of LmigOBP1 **(A)**, OasiOBP3 **(C)** and SgreOBP6 **(E)** varies greatly at a global surface scale as well as on the local C-terminal surface scale. **(B,D,F)** Depiction of the interior cavity (left) which is bordered by C-terminal domain (highlighted in purple) and the corresponding electrostatic potential map (right). The assumed enlarged interior cavity in LmigOBP1 **(B)** relative to OasiOBP3 **(D)** and SgreOBP6 **(F)** is outlined with a white dash line. Electrostatic potential was calculated in the range of −6 to 6 kT/e and was presented as blue-red hue gradient. Blue, negative potential; red, positive potential; k, Boltzmann's constant; T, temperature; e, charge of an electron.

**Figure 5:**
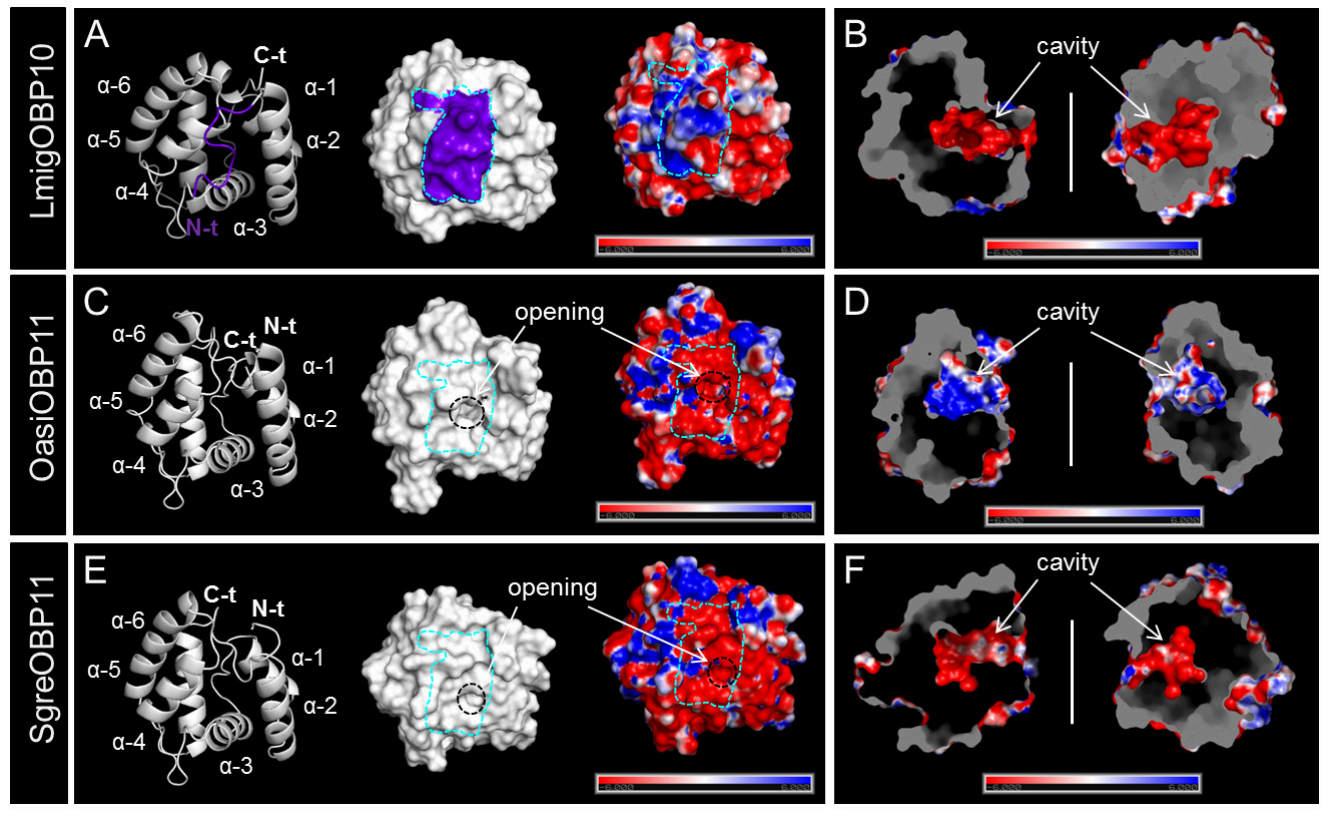
The surface topologies and interior cavities of subfamily II-A OBPs. **(A,C,E)** Comparison of the backbone structures, surface topologies, and surface potentials of LmigOBP10, OasiOBP11, and SgreOBP11 which represent the three different ortholog groups in subfamily II-A. Left: the prolonged N-terminus in LmigOBP10 (see Figure S4B) was predicted to form a short coiled-coil shown on the backbone structure (highlighted in purple, **A**), but was absent from OasiOBP11 **(C)** and SgreOBP11 **(E)**. Middle: the N-terminal domain of LmigOBP10 was plotted on the surface and sketched by a cyan dash line **(A)**. The N-terminal domain of LmigOBP10 was labeled on the same surface position for OasiOBP11 **(C)** and SgreOBP11 **(E)**. The visible opening structure is denoted by a black circle for OasiOBP11 **(C)** and SgreOBP11 **(E)**. Right: a map of electrostatic potential on the molecular surface. Generally similar electrostatic potential pattern is observed among LmigOBP10 **(A)**, OasiOBP11 **(C)** and SgreOBP11 **(E)**. **(B,D,F)** A symmetric presentation of the interior cavity with the electrostatic potential. Electrostatic potential was calculated in the range of −6 to 6 kT/e and was presented as blue-red hue gradient. Blue, negative potential; red, positive potential; k, Boltzmann's constant; T, temperature; e, charge of an electron.

Multiple sequence alignment of subfamily I-A OBPs revealed a striking variation on the C-terminal domain (Figure S4). It is known that LmigOBP1 has a prolonged C-terminus with ~17 amino acids to form a seventh α-helix (Zheng et al., 2015). In contrast, the C-terminus in OasiOBP3 and SgreOBP6 is shortened to a 7 amino acids motif and most likely constitute a coiled-coil strand instead of a seventh α-helix (Figure 4); a groove emerged on the collapsed surface due to the shortened C-terminus. The electrostatic potential pattern varies greatly at a global surface scale as well as on the local C-terminal surface scale (cyan dash line, Figures 4A,C,E). Another striking structural difference is the enlarged cavity of LmigOBP1 bordered by the prolonged C-terminus, whereas the cavity for the other two counterparts, representative of different ortholog groups shrinks to some extent (white dash line, Figures 4B,D,F). Unlike subfamily I-A, the multiple sequence alignment of subfamily II-A OBPs exhibited an aligned C-terminus but an unaligned N-terminus, namely, an extra extension of a 9–10 amino acids motif in the LmigOBP10 ortholog group (Figure S4). Correspondingly, this alteration was predicted to result in a coiled-coil structure on the N-terminal domain for LmigOBP10; at the same surface position, an opening structure was observed on its two counterparts, the OasiOBP11 and SgreOBP11 (Figures 5A,C,E). Apart from that, the surface electrostatic potential profile seems to vary slightly, both at the global surface scale and at the local N-terminal surface scale (cyan dash line, Figures 5C,E), regardless of the extra N-terminal coil present on LmigOBP10. However, the interior cavity could be enriched with negative potentials (LmigOBP10 and SgreOBP11, Figures 5B,F), or with positive potentials (OasiOBP11, Figure 5C).

### Topographic expression patterns of SgreOBPs from subfamily I-A and II-A

**Figure 6:**
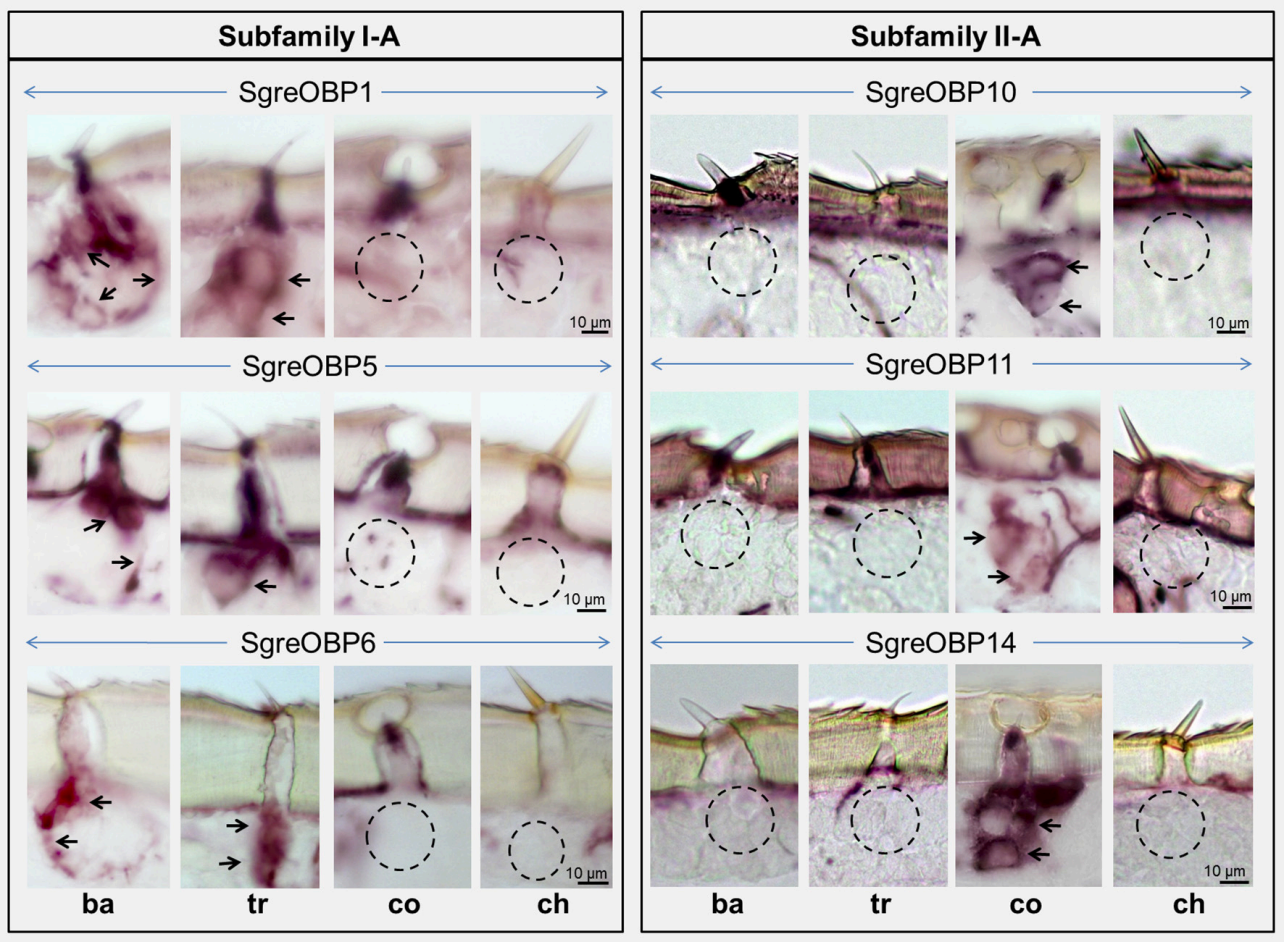
Sensilla-specific expression of subfamily I-A and II-A OBPs in the antenna of *S. gregaria*. Antisense riboprobes which specifically target the SgreOBPs were used to visualize the appropriate structures by means of chromogenic *in situ* hybridization (ISH). SgreOBP1, SgreOBP5, and SgreOBP6 are representing three different ortholog groups of subfamily I-A, whereas SgreOBP10, SgreOBP11, and SgreOBP14 are representing three different ortholog groups of subfamily II-A. Labeling obtained with probes for subfamily I-A SgreOBPs was restricted to sensilla basiconica (ba) and sensilla trichodea (tr), but was absent in sensilla coeloconica (co) and sensilla chaetica (ch). Labeling obtained with probes for subfamily II-A SgreOBPs was detected only in sensilla coeloconica (co), but was absent in the other three sensillum types. Black arrows indicate the visible OBP labeling while black circles denote the absence of OBP labeling.

To approach this question, whether locust subfamily I-A and II-A OBPs may be expressed in different sensillum types and different cells, we set out to unravel the expression patterns of SgreOBPs from the two locust subfamilies in sensilla on the antenna, the major olfactory organ. By adopting RNA *in situ* hybridization (ISH) on antennal sections using specific OBP probes, we acquired a strikingly sensilla-specific expression pattern for SgreOBPs in the two subfamilies. For SgreOBP1, SgreOBP5 and SgreOBP6, the members of subfamily I-A, we found alike expression in the cells of both sensilla basiconica and sensilla trichodea (Figure 6). In contrast, none of the subfamily I-A SgreOBPs was expressed in sensilla coeloconica or sensilla chaetica. Conversely, for members of subfamily II-A SgreOBPs, namely, SgreOBP10, OBP11, and OBP14, the expression was found to be restricted to the cells of sensilla coeloconica; there was no evidence for an expression in cells of any other sensillum type (Figure 6). The notion that a similar expression pattern is conserved for orthologous OBPs from other locust species is supported by the finding that LmigOBP1 is specifically expressed in sensilla basiconica and sensilla trichodea of *L. migratoria* (Jin et al., 2005), alike its ortholog in *S. gregaria*, the SgreOBP1.

Thus, an apparent sensilla-specific expression pattern for each locust OBP subfamily emerged. To extend and specify this aspect, the expression of OBP subtypes was compared with the expression of sensilla-specific receptor types. The odorant receptor co-receptor Orco and the ionotropic receptor (IR) type IR8a are ubiquitous co-receptors expressed in insect OSNs, either together with ligand-specific ORs or with IRs, and are considered as general markers for sensilla basiconica/sensilla trichodea and sensilla coeloconica, respectively (Yang et al., 2012; Guo et al., 2013). As a marker specific for distinct sensilla trichodea, the expression of the sensilla-specific receptor type OR3 in *S. gregaria* was monitored (Pregitzer et al., 2017). We designed riboprobes labeled by either Dig or Bio, which specifically targeted the distinct sensory neuron markers and SgreOBPs of the two subfamilies. Subsequently, two-color fluorescent *in situ* hybridization (FISH) experiments were performed to visualize the expressing cells (Figure S5). The results indicated that SgreOBPs of subfamily I-A are expressed in cells located in sensilla basiconica; these cells extended cytoplasmic processes and enclosed clusters of Orco expressing neurons. Similarly, SgreOBPs of subfamily I-A were found to be expressed in cells located in sensilla trichodea, as characterized by their close association with OR3 expressing OSNs. In the sensilla coeloconica, characterized by the IR8a-positive neurons, the neurons were found to be engulfed by cells which express OBPs of the subfamily II-A.

**Figure 7:**
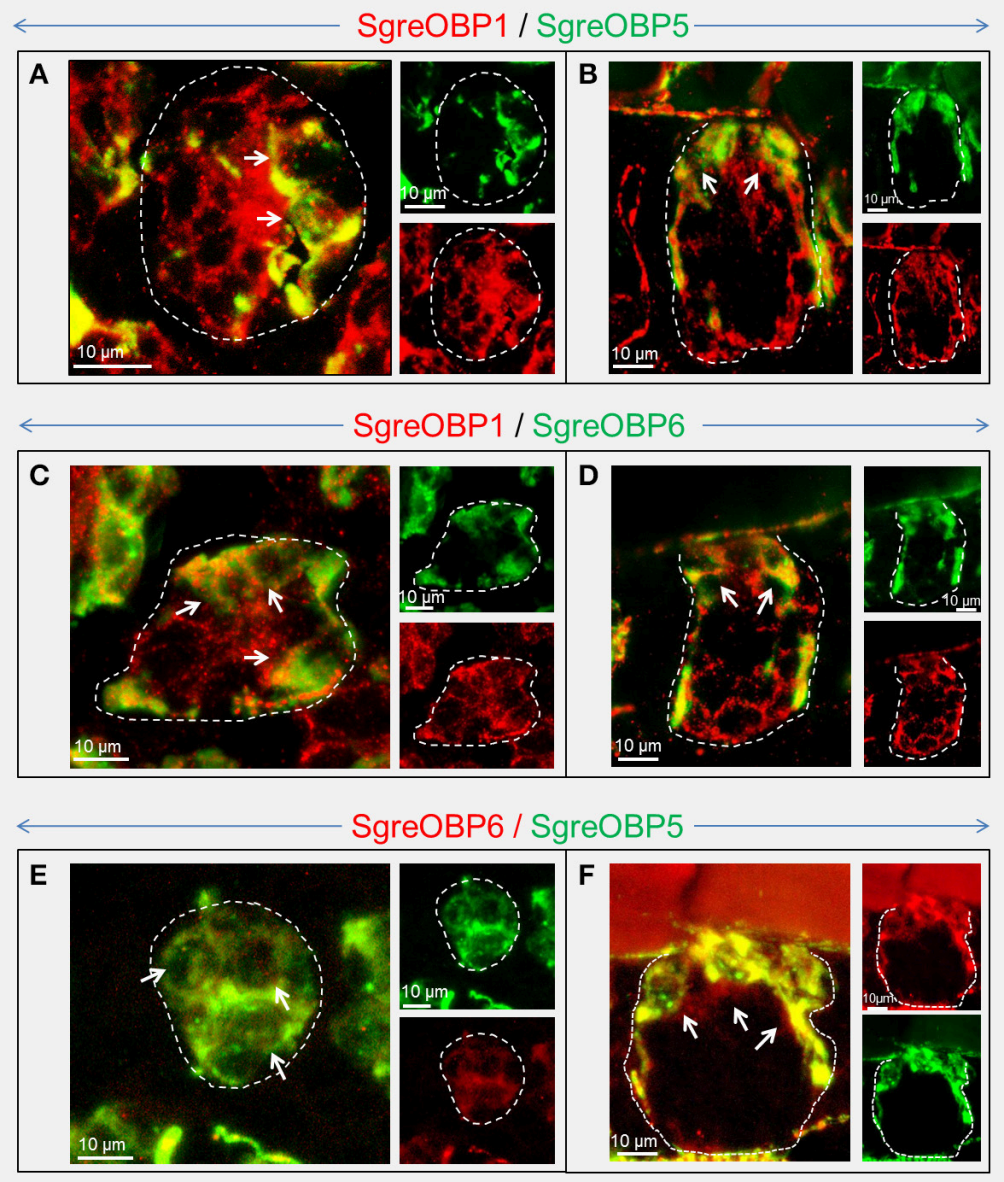
Visualization of cells expressing distinct subtypes of subfamily I-A SgreOBPs. The confocal images show the co-localization of three SgreOBPs from subfamily I-A in the cellular compartment of a sensillum basiconicum. Cells expressing distinct subtypes of subfamily I-A SgreOBPs were visualized by two-color FISH employing subtype specific antisense riboprobes. Confocal images of the overlaid green and red fluorescence channel are shown at higher magnification on the left, the red and green fluorescent channels are shown separately at lower magnification on the right. Cells that are apparently assigned to the cell-cluster belonging to one sensillum basiconicum are outlined in a white dash line. Cells that co-express two distinct OBP subtypes are indicated by white arrows. **(A,C,E)** A horizontal perspective of the superficial cellular layer close to the cuticle is shown where the cytoplasmic processes exhibited by subfamily I-A SgreOBP-positive cells are less likely to be visualized. **(B,D,F)** A longitudinal perspective of a deep layer beneath the cuticle is shown where the cytoplasmic processes are likely to be visualized. **(A–D)** It is noted that a smaller number of cells are labeled in green compared to the number of cells labeled in red.

Although, our data demonstrated that SgreOBPs from different ortholog groups in each subfamily are expressed in the same sensillum type, it remained unclear to what extent they are expressed in the same set of sensilla and whether they are co-expressed in the same cells within a distinct sensillum. To resolve this question, we performed two-color FISH on sections through the antenna of *S. gregaria* using riboprobes targeting SgreOBPs from different ortholog groups. The results for SgreOBPs in subfamily I-A indicate that SgreOBP1 was expressed in a cell population present in almost all basiconic and trichoid sensilla, whereas SgreOBP5 and SgreOBP6 were expressed only in a much smaller subset of cells than SgreOBP1 in the same sensillum (Figure 7). These differences became apparent in both horizontal sections giving a view onto superficial cellular layer (no cytoplasmic process expected, Figure 7A) as well as in longitudinal sections which allowed a view into deeper layers (cytoplasmic process expected, Figure 7B) of the antenna. Unlike SgreOBP1-positive cells which could be visualized both at the superficial and the deeper cellular layer, most of SgreOBP5- and SgreOBP6-positive cells appeared to be restricted to the superficial cellular layer close to the cuticle; slim cytoplasmic processes stretched to deeper cellular layers. Incidentally, there was evidence that SgreOBP5 and SgreOBP6 were expressed in the same set of cells of a sensillum (Figures 7E,F).

**Figure 8:**
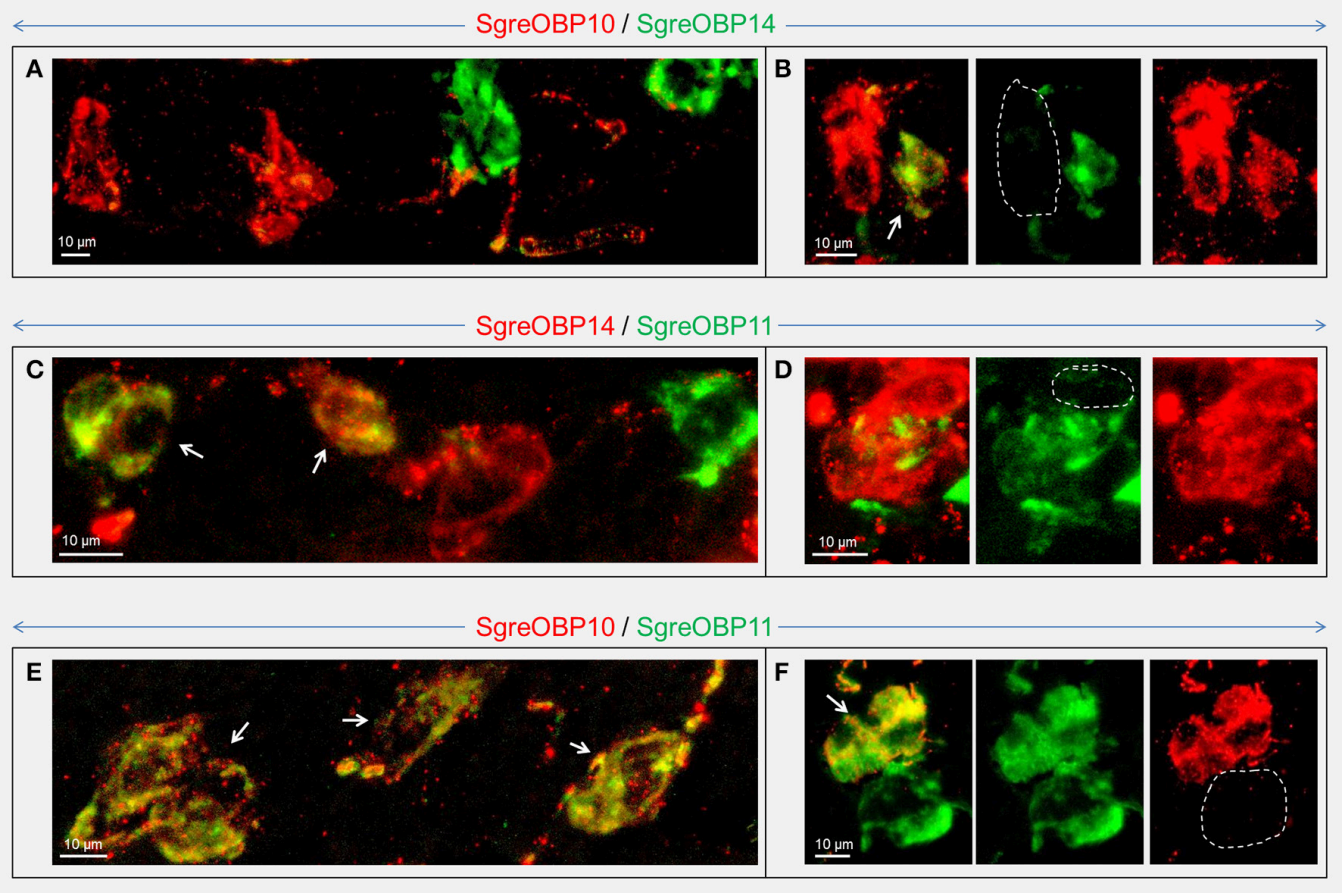
Visualization of cells expressing distinct subtypes of subfamily II-A SgreOBPs. Cells expressing distinct subtypes of subfamily II-A SgreOBPs were visualized by two-color FISH employing combinations of subtype specific antisense riboprobes. The dash line indicates the absence of OBP labeling at the particular area. **(A)** A representative confocal image demonstrating expression of SgreOBP10 and SgreOBP14 in separate cells. **(B)** Rarely cells could be observed that co-expressed SgreOBP10 and SgreOBP14 (white arrows). **(C,D)** For the combination of SgreOBP14 and SgreOBP11 co-expression (white arrows) was observed at a similar rate as a separate expression of the two OBPs in different sensilla. **(E)** SgreOBP10 and SgreOBP11 were frequently found to be co-expressed in the same cells (white arrows). **(F)** Only few cells were detected that selectively expressed only one of the OBP subtypes. **(B,D,F)** Confocal images show the overlaid fluorescence channels (left) as well as the separated green and red fluorescence channels (middle and right) on the same magnification.

In contrast to the subfamily I-A, for subfamily II-A we did not find any evidence for an OBP subtype that was ubiquitously expressed in coeloconic sensilla (Figure S5). This result has led to the notion that particular OBP members of subfamily II-A may be specifically expressed in subsets of coeloconic sensilla. In fact, we frequently observed that expression of SgreOBP10 and SgreOBP14 were restricted to different cells in sensilla coeloconica (Figures 8A,B). For the subtypes SgreOBP11 and SgreOBP14 a co-expression in the same cells or expression in different cells were observed at a similar rate (Figures 8C,D). For the subtypes SgreOBP10 and SgreOBP11 it was frequently observed that they were co-expressed in the same cells (Figure 8E), indeed, more often than an expression in different cells (Figure 8F). Moreover, we verified the spatially separated expression of SgreOBPs from subfamily I-A and II-A (Figure S6), consistent with the results in Figures 6, 7. Taken together, the results unravel a characteristic subfamily-dependent cellular expression pattern for different OBP subtypes.

## Discussion

The complex behavior of locust species, including the unique switch between a solitarious phase and a gregarious phase, is strongly based on a sophisticated chemical communication system (Pener and Yerushalmi, 1998; Hassanali et al., 2005; Wang and Kang, 2014). Great efforts have been made to unravel the chemical cues and underlying chemosensory mechanisms in mediating locust enigmatic behavior (Heifetz et al., 1996; Anton et al., 2007). Out of these efforts, a variety of olfactory genes, including gene families encoding odorant receptors and candidate pheromone receptors have recently been identified (Guo et al., 2011; Wang et al., 2015; Pregitzer et al., 2017). Since much less was known about their counterparts which deliver the olfactory signal molecules to the receptors, the OBPs, this study was concentrating on a systematic analysis of locust OBPs with respect to their molecular evolution as well as on an evaluation of predicted protein structures for OBP subtypes and their expression pattern in stinct sensillum types.

The in-depth analysis of locust OBP sequences uncovered the presence of both common and specific amino acid motifs (Figure 2). The common motifs adequately recapitulate sequence information in most of the locust OBPs, while specific motifs selectively represent locust OBP subfamilies which may contribute to the clustering of sequences on the phylogenetic tree (Figure 1). The mixed common and specific motif profile is reminiscent of the findings that selection regimes may vary among different sequence domains (Policy and Conway, 2001; Sawyer et al., 2005). The subfamily specific motifs define sequence domains that apparently withstand diversifying selection constraints, presumably shaped by the sensilla environment, including their likely interplay-partner, the endogenous receptor types (Figure S5). In contrast, the common motifs define sequence domains that appear to share similar stabilizing selection constraints, presumably required for the maintenance of the common globular structures of the proteins (Pelosi et al., 2017), or for retaining the conserved ligand binding sites (Yu et al., 2009).

The four locust species tackled in this study differ significantly in their geographic distribution. While *S. gregaria* (the desert locust) occurs in Africa, the Middle East and Asia and *L. migratoria* (the migratory locust) in Africa and Asia, but also in Australia and New Zealand, the locusts *O. asiaticus* and *C. kiangsu* (the yellow-spined bamboo locust) appear to live locally in North China and South China. Nevertheless, a molecular and evolutionary stabilized status can be assigned to locust OBP subfamily I-A and II-A that appear to be subject to purifying selection pressure (Figure 3C), indicative for conserved chemosensory roles. In addition, the chemosensory adaptation to different habitats supposedly implies positive selection constraints (Cicconardi et al., 2017), and several of the locust OBPs appear to reflect such a selection regime (Figure 3C).

For the locust OBP subfamily I-A, the selective expression in two distinct sensillum types, sensilla basiconica, and sensilla trichodea, appears to be a characteristic hallmark (Figure 6 and Figure S5). This feature is also found for OBPs from other species, which are orthologous of locust OBPs subfamily I-A (Figure 3D). For example, in *Drosophila melanogaster*, most of the subfamily I-A orthologous OBPs are associated with sensilla basiconica and sensilla trichodea, similar to their locust counterparts. It was found that DmelOBP83a and DmelOBP83b were associated with sensilla basiconica and sensilla trichodea, while DmelOBP69a and DmelOBP76a seemed to be restricted to sensilla trichodea (Larter et al., 2016). However, for a few orthologous OBPs such as DmelOBP56d an extra sensillar expression has been reported (Larter et al., 2016). The concept of a sensilla-specific expression pattern for orthologous OBPs of locust subfamily I-A is also supported by the finding in the moth *Manduca sexta*, where two orthologous OBPs of locust subfamily I-A, named MsexABP2 and MsexABPx, are specifically expressed in sensilla basiconica (Nardi et al., 2003). Since the Orthopteran locust species emerged at a much earlier stage than the moth and fly species during the insect species divergence (Vieira and Rozas, 2011; Vogt et al., 2015), it is conceivable that a dual expression of subfamily I-A OBPs in both sensilla basiconica and sensilla trichodea may represent an ancestral status. In insect species like moths and flies, which emerged later in evolution, some OBP subtypes may have evolved towards a more specific function and expression in either sensilla basiconica or sensilla trichodea (Maida et al., 2005; Larter et al., 2016).

Our analysis suggests that the locust OBPs of subfamily II-A and their orthologous OBPs in other species have originated from a common ancestor (Figure S3), and may share a sensilla coeloconica specific expression pattern (Figure 6, Figure S5). In *Drosophila melanogaster*, DmelOBP84a, the only orthologous OBP of locust subfamily II-A is actually among the few OBPs that have been reported to be specifically expressed in sensilla coeloconica (Larter et al., 2016). Interestingly, the gene encoding OBP84a is retained in most, if not all, *Drosophila* species genomes (Cicconardi et al., 2017). Moreover, the OBP84a ortholog group in *Drosophila* species withstands apparent purifying selection pressure (Vieira et al., 2007) and converges onto a segregated phylogenetic clade (Cicconardi et al., 2017), which is very similar to the locust OBP subfamily II-A. These molecular and phylogenetic commonalities may point to some similarities with regard to their functional roles. In this regard, it is interesting to note that single sensillum recordings from sensilla coeloconica of locust, flies and moths have revealed a response spectrum confined to certain ecologically important odorants, including organic acid, amines and plant derived odorants (Pophof, 1997; Ochieng and Hansson, 1999; Yao, 2005). Thus, it will be of particular interest to unravel a potential role of locust subfamily II-A OBPs and their orthologs in other species for the detection of cognate odorants in sensilla coeloconica. While concentrating on OBPs of subfamily II-A, we are aware that sensilla coeloconica may also comprise OBPs of other phylogenetic clades.

Unlike DmelOBP84a, which is broadly expressed in almost all sensilla coeloconica (Larter et al., 2016), the OBPs of the locust subfamily II-A are expressed in sensilla coeloconica in a combinatorial mode (Figure 8). This is in line with the previous finding that different subsets of sensilla coeloconica in *S. gregaria* showed individual response spectra to a repertoire of odorants (Ochieng and Hansson, 1999), suggesting a sensilla-specific response spectrum and sensilla-specific repertoire of odorant sensing proteins. Thus, it is conceivable that a distinct combination of OBPs in a sensillum coeloconicum (Figure 8) may correlate with particular endogenous IR types.

Although amino acid sequences of OBPs can be highly divergent, the folding of proteins forming a hydrophobic pocket is well conserved across insect species; in fact to date the structures of more than 20 OBPs have been solved by X-ray crystallography and/or nuclear magnetic resonance (NMR) spectroscopy (Pelosi et al., 2017). The results of these studies revealed that the C-terminal domain, especially the length of the C-terminus has important implications on the mechanism of ligand-binding (Tegoni et al., 2004). Long C-terminus apparently enter the binding pocket and determine the shape of the cavity (Sandler et al., 2000), medium-length C-terminus act as a lid covering the entrance to the binding pocket (Lartigue et al., 2004). In view of these findings, simulation of the putative tertiary structures of locust OBPs revealed some interesting features. The three ortholog groups of subfamily I-A significantly differ in their C-terminal domain. LmigOBP1 and its orthologs have a long (17 aa) C-terminus, long enough to form an extra α-helix and thus affecting the shape of the cavity (Figure 4, Figure S4); other two ortholog groups have both a medium size C-terminus (7 aa), however, significantly different in the amino acid sequence. These observations may suggest significant differences in the mechanisms of OBP/ligand interaction among the three ortholog groups in subfamily I-A.

The results of this study indicate that in a considerable number of sensilla at least two OBP subtypes are co-expressed (Figures 7, 8). This is of particular interest, since hetero- and homo-dimerization of OBPs have been reported *in vitro* (Andronopoulou et al., 2006), which is accompanied by a set of conformational changes (Wogulis et al., 2006; Mao et al., 2010). Although the underlying mechanisms are still elusive, there is evidence that electrostatic interaction at short range forming the salt bridges may contribute to specific protein-protein interaction (Sheinerman et al., 2000; Kumar and Nussinov, 2002). In locusts, the patch of charged residues buried on the OBP-interface (Figures 4, 5) is likely to provide hot spots for protein-protein interactions. In addition, changes of the OBP tertiary structure has been demonstrated as a consequence of pH changes in the environment (Zubkov et al., 2005; Pesenti et al., 2008). This notion may also fit for locust OBPs since an intermingled distribution of both negative and positive charged residues was observed by elucidating a map of electrostatic potentials (Figures 4, 5). The presence of multiple OBP subtypes and their possible interaction may have functional implications for the binding capacity of the olfactory system. In fact, recent binding assays have shown that in the presence of two OBPs the binding affinity to cognate ligands altered considerably compared to the binding characteristics of a single OBP type (Qiao et al., 2011; Sun et al., 2016). This notion may be particular relevant with respect to sensilla basiconica of locusts, which house up to 50 sensory neurons responding to a variety of different odorants (Ochieng et al., 1998; Ochieng and Hansson, 1999), and the fact that the number of OBP genes is much smaller than the size of the OR gene family in locusts, encoding more than 140 ORs in *L. migratoria* (Wang et al., 2014) and at least 120 ORs in *S. gregaria* (Pregitzer et al., 2017). The selective sensilla expression pattern implies that a small number of OBP subtypes are present in the sensillum lymph (Figure 7, Figure S6). Assuming that each OBP subtype has distinct ligand specificity, the mixture may provide a much broader binding spectrum. A possible combinatorial mode of OBP participation in locust olfaction is an interesting aspect for future studies.

## Author contributions

HB, JK, XJ, and PP conceived the study. XJ conducted the experiments. HB, JK, XJ, and PP interpreted the results. XJ and PP drafted the preliminary manuscript. HB and JK refined and approved the final manuscript.

### Conflict of interest statement

The authors declare that the research was conducted in the absence of any commercial or financial relationships that could be construed as a potential conflict of interest.
