# Supplementary material for: Distinct Subfamilies of Odorant Binding Proteins in Locust (Orthoptera, Acrididae): Molecular Evolution, Structural Variation, and Sensilla-Specific Expression
Source: Front Physiol. 2017 Sep 26;8:734. doi: 10.3389/fphys.2017.00734 (PMC5623057; doi:10.3389/fphys.2017.00734)
Supplement: Supplementary file 1 [file Presentation1.PDF]

Supplementary material

Distinct subfamilies of odorant binding proteins in locust (Orthoptera, Acrididae): molecular evolution, structural variation and sensilla-specific expression

Xingcong Jiang[1], Jürgen Krieger[2], Heinz Breer[1] and Pablo Pregitzer[1*]

[1] Institute of Physiology, University of Hohenheim, Stuttgart, Germany

[2] Institute of Biology/Zoology, Department of Animal Physiology, Martin Luther University Halle-Wittenberg, Halle, Germany

Correspondence*

Pablo Pregitzer
University of Hohenheim, Institute of Physiology (230), Stuttgart, Germany, phone: +49 711 459 22270, fax: +49 711 459 23726, email: p_pregitzer@uni-hohenheim.de

Supplementary figures and figure legends

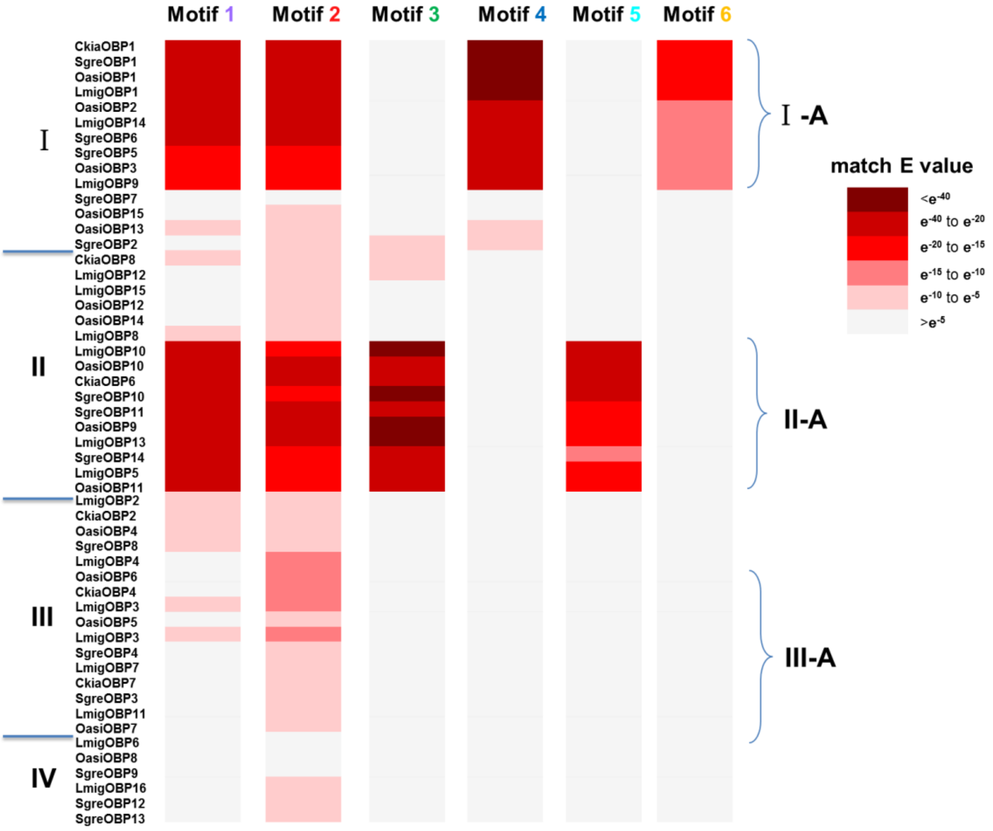

Figure S1. Map of match E-values of six consensus amino acid motifs related to the locust OBP repertoire. The six identified consensus motifs from **Fig. 2** were used to target locust OBP sequences individually to obtain the motif match E-value. The match E-value assesses statistical significance of the consensus motif towards a particular sequence based on its log likelihood level and the occurrence frequencies of background amino acid. The gradually enhancing red represents better matching of a motif related to the targeted sequence with a match E-value below e^{-5} (default statistical significant threshold). The order of sequences as well as the division of OBP families (I - IV) and subfamilies (I-A, II-A and III-A) is guided by the phylogenetic tree topology in **Fig. 1**.

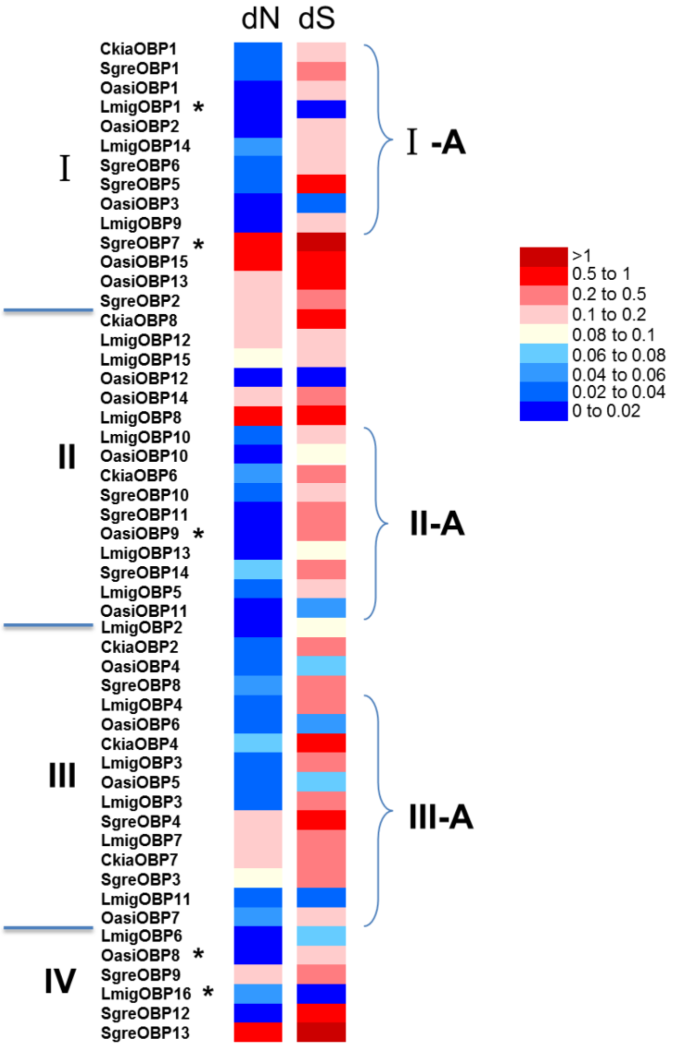

Figure S2. dN and dS rates obtained for the locust OBP repertoire. OBPs with the calculated dN and /or dS =0 (symbolized with *) were excluded for dN, dS and ω rates (dN /dS) analyses. Order of sequences is guided by the phylogenetic tree in **Fig. 1**.

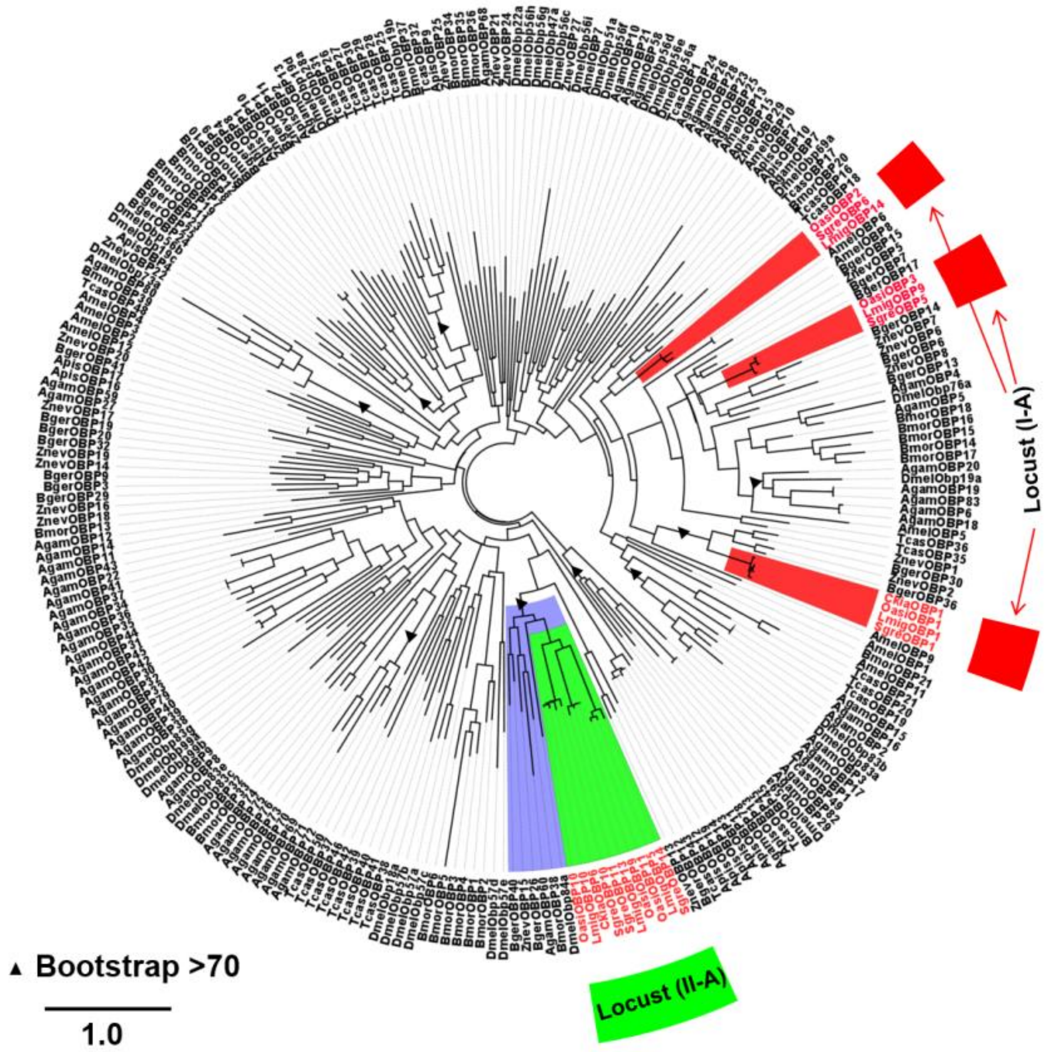

Figure S3. Phylogenetic relationship between locust OBPs and OBPs of 8 other insect species. Phylogenetic analysis utilizing locust subfamily I-A and II-A OBPs and classic OBPs from 8 other insect species, namely, *Anopheles gambiae* (Agam, Diptera), *Apis Mellifera* (Amel, Hemiptera), *Drosophila melanogaster* (Dmel, Diptera), *Tribolium castaneum* (Tcas, Coleoptera), *Acyrthosyphon pisum* (Apis, Hemiptera), *Bombyx mori* (Bmor, Lepidoptera) (Vieira and Rozas, 2011), *Blattella germanica* (Bger, Blattaria) (Niu et al., 2016) and *Zootermopsis nevadensis* (Znev, Isoptera) (Terrapon et al., 2014). The phylogenetic tree was generated using the maximum likelihood algorithm, supported by 1000 bootstrap replicates. Major internal branches with bootstrap support above 70% are denoted as black triangle. The names of locust subfamily I-A and II-A are written in red characters. The branches of I-A and II-A OBPs are highlighted by red and green shading, respectively. The common branch from which locust subfamily II-A and their orthologs were diverged is shaded in purple. The scale bar represents one amino acid substitution per site.

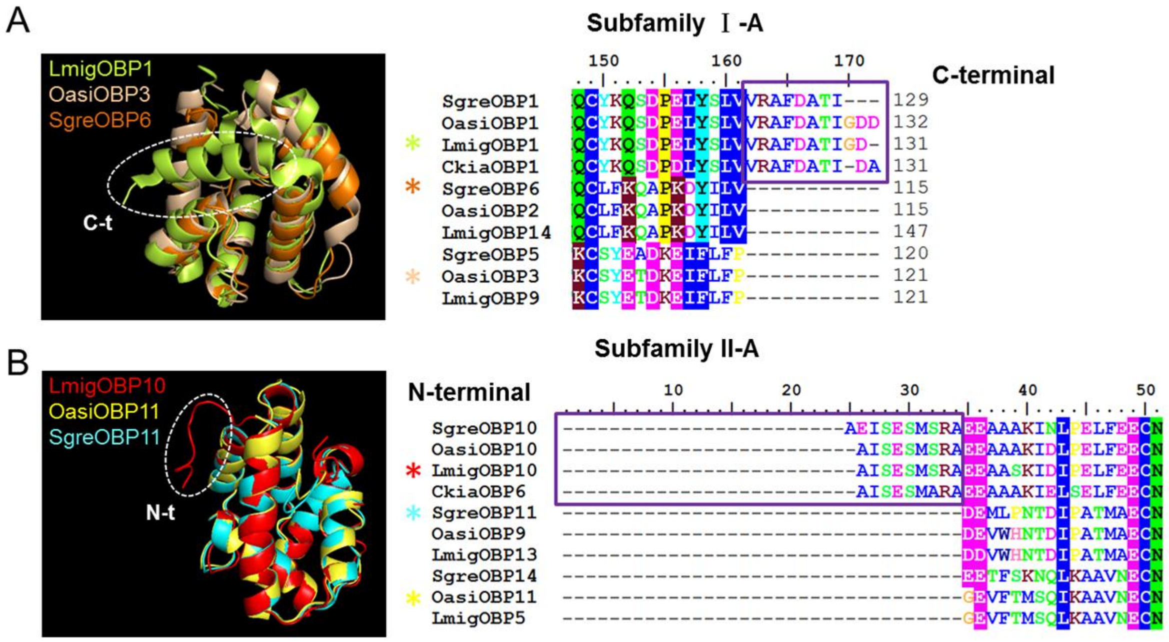

C

	Subfamily I-A // LmigOBP1		Subfamily II-A // LmigOBP1		
	OasiOBP3	SgreOBP6	LmigOBP10	OasiOBP11	SgreOBP11
RMSD	1.52	1.27	2.59	3.11	2.69

Figure S4. Multiple sequence alignment and superimposition of simulated OBP structures to LmigOBP1. Superimposed backbone structures (left) and terminal sequence alignments (right) in (**A**) and (**B**) were made with OBPs representing three different ortholog groups from subfamily I-A and II-A, respectively. (**A**) Analysis of the C-terminal domain of subfamily I-A OBPs. The prolonged C-terminal domain in LmigOBP1 and its orthologs (boxed area in the alignment) forms a seventh α-helix (Zheng et al., 2015) (dash line oval in the superimposition), which is presumably absent in the other two ortholog groups (represented by OasiOBP3 and SgreOBP6), due to the shortened C-terminal domain that may form a coiled-coil structure (boxed area in the alignment). (**B**) Analysis of the N-terminal domain of subfamily II-A OBPs. A prolonged N-terminal domain in LmigOBP10 and its orthologs (boxed area in the alignment) was predicted to form a short coiled-coil (dash line oval in the superimposition) ahead of the first α-helix. (**C**) RMSD scores obtained by superimposing simulated OBPs in subfamily I-A and subfamily II-A to LmigOBP1, the one that has been resolved of crystal structure.

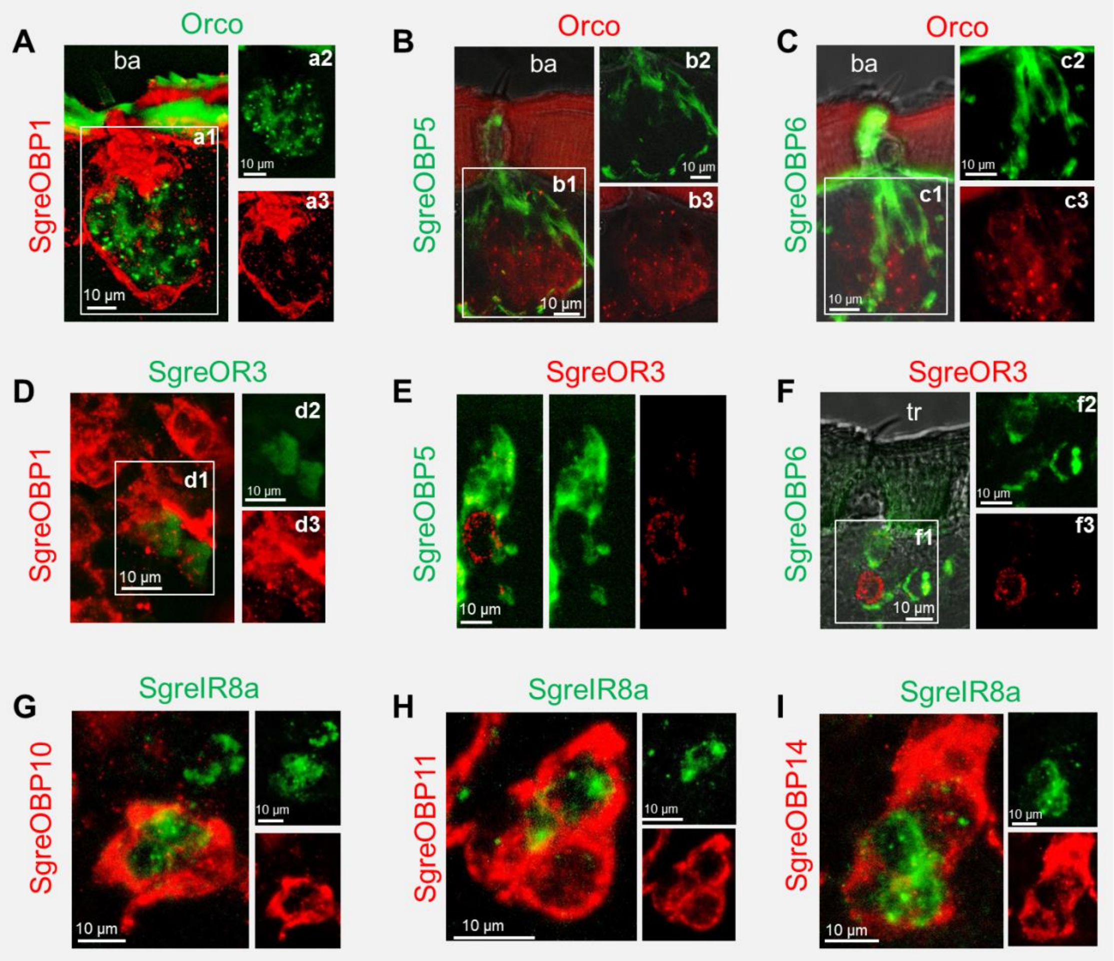

Figure S5. Cells expressing subfamily I-A and II-A SgreOBPs associate with different types of sensory neurons. Gene-specific antisense riboprobes labelled with either biotin (green) or digoxigenin (red) were used to visualize the appropriate structures by means of two-color fluorescent *in situ* hybridization (FISH). Labeled probes for Orco and IR8a, the ubiquitous co-receptor of ORs and IRs, were used as molecular markers for sensilla basiconica and sensilla coeloconica, respectively. A labeled probe for SgreOR3 was used as a specific marker for distinct sensilla trichodea. (**A**-**F**) Cells expressing SgreOBPs of locust subfamily I-A are associated with OR expressing cells. SgreOBP1, SgreOBP5 and SgreOBP6 expressing cells were found to extend cytoplasmic processes and surround the ORNs in sensilla basiconica and sensilla trichodea. The green (**a**2-**d**2 and **f**2) and red (**a**3-**d**3 and **f**3) fluorescence channels are shown separately for the boxed area indicated in pictures of the overlaid fluorescence channels (**a**1-**d**1 and **f**1). (**G**-**I**) Cells expressing SgreOBPs of subfamily II-A associate with IR expressing cells. SgreOBP10, SgreOBP11 and SgreOBP14 expressing cells were observed to tightly envelope IR8a-positive sensory neurons, which are housed in sensilla coeloconica. Confocal images of the separated fluorescence channels are shown at a reduced-size (right) next to the overlaid fluorescence channels (left).

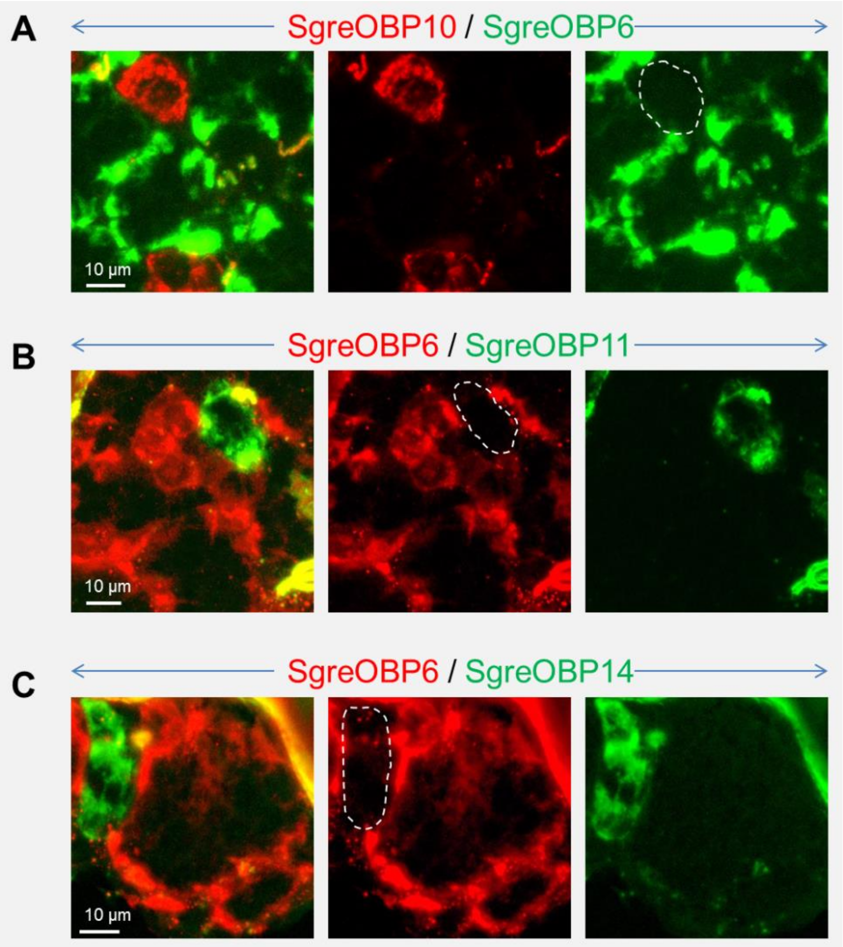

Figure S6. Spatial segregation of cells expressing subfamily I-A and II-A SgreOBPs. Labeling of specific OBPs was visualized via two-color FISH. Absent labeling of subfamily II-A SgreOBPs at the particular area was sketched in white dash line. (**A**-**C**) Labeling of subfamily II-A SgreOBPs, SgreOBP10, OBP11 and OBP14 did not overlap with that of SgreOBP6, representative of subfamily I-A SgreOBPs. Confocal images show the overlaid fluorescence channels (left) as well as the separated red and green fluorescence channels (middle and right) on the same magnification.

Reference

Niu, D. J., Liu, Y., Dong, X. T., and Dong, S. L. (2016). Transcriptome based identification and tissue expression profiles of chemosensory genes in *Blattella germanica* (Blattaria: Blattidae). *Comp. Biochem. Physiol. - Part D Genomics Proteomics* 18, 30–43. doi:10.1016/j.cbd.2016.03.002

Terrapon, N., Li, C., Robertson, H. M., Ji, L., Meng, X., Booth, W., et al. (2014). Molecular traces of alternative social organization in a termite genome. *Nat. Commun.* 5. doi:10.1038/ncomms4636

Vieira, F. G., and Rozas, J. (2011). Comparative genomics of the odorant-binding and chemosensory protein gene families across the arthropoda: origin and evolutionary history of the chemosensory system. *Genome Biol. Evol.* 3, 476–490. doi:10.1093/gbe/evr033

Zheng, J., Li, J., Han, L., Wang, Y., Wu, W., Qi, X., et al. (2015). Crystal structure of the *Locusta migratoria* odorant binding protein. *Biochem. Biophys. Res. Commun.* 456, 737–742. doi:10.1016/j.bbrc.2014.12.048

Sequence information (Accession Number in bold)

>LMIGOBP1 **DQ208934.1**
ATGTGGGCCCGCCTCAACGACTGCGCCGCTCTGCTGCTGCTGCTGGCCGCCGCCG
CCCGCGCCTGGGACGTCAACATGAAACTTACTGGGCGCATTATGGATGCTGCAAA
AGAAGTGGACCACACATGCCGCTCATCTACTGGGGTTCCAAGAGACATGCTCCAT
AGATATGCTGAAGGTCAAACTGTAGATGATGATGATTTCAAGTGTTACCTGAAGT
GTATTATGGTTGAGTTTAATTCACTCTCAGATGATGGAGTTTTTGTTTTAGAAGAA
GAATTAGAAAATGTTCCTCCAGAAATAAAGGAAGAAGGCCATAGGGTTGTACAC
AGCTGCAAACACATAAATCATGATGAAGCTTGCGAAACAGCTTACCAGATCCATC
AGTGCTATAAACAGAGTGATCCTGAGTTGTACAGCCTGGTAGTTCGTGCATTTGA
TGCAACCATTGGTGAC
>LMIGOBP2 **ACR39388.1**
TGGATCCCCGGGCTGAGGAATTCGAATTCCGACATGAGGACCTCCGCAGCCGCAG
CCGCCACCGGACCAGCGTTCCTCGTGCTCGCCGCCGTCGCATCGGCGATGGAGAT
GACACCAGAATTCATGGAGATAGTTAACAAGTGCAAGACAGAACATGAACCCAC
CGAAGATGAGCTGAAGGGGATGATGGCGTTGAAAGTTCCAGAGAGTGCCAACGG
CAAGTGCTTCATGGGGTGCGTTCTACAGGAAATTGGAGTGGTAAAGGATGGCAA
GTTCGACAAGGAGGAGGCGAAGAAACACGCCGCTTCCAAGATGACTGACAAAGA
CGAACTGGAGAAGCACATGCAGCTCATTGAGAAATGTAGCCAAGAAGTTGGTGG
TGAGACGGACTCTCGCGGAATCGGCCCCAAACTCATGGAGTGCATCAAGCAGTTT
GCACCAGAGTTTGACATTGCCCTCCCGCAGCAACCGTCAGAA
>LMIGOBP3 **ACR39392.1**
GACGCGGAGAAGATGAAGGAAGCCGTAGACAAATGCAAGGCGTCCGAAAACCT
GGATAGCTTGGACGGCCTTAAGAGTAGCAAGTCACCCTCCACCGAAGAAGAGAA
GTGCTTCATTGGCTGTCTGATGATGGATATGAAATTGTTGTCTTCAGATGGGCAGT
ACGATGCCGCCAGCACGAAGGACATGATCAACAACTGCGAGTACCTCAAGGACA
AACCCGACGAGAAGAGCGTCGCCCTGGAGGTGGCCGACGACTGCGCCGGCAAAG
CTACTGGCTGCAGCGGCCACTGCGAATGCGGACCCAAGGCGGTTGGCTGCCTGAT
AAAGGGCATGGTTGACAAGGGCTACGAGGAATCGTTCGCAAGAATTGACAAAAT
GTTAGAAAAACTTGATGAT
>LMIGOBP4 **AEV45802.1**
ATGGGCACCGCCGTAGCCGCTGCAGTTCTCCTACTGGTTGCTGTCGCCAACGCCG
AGGACTCGTTGATGGAAATAGTCATCCGGGAGGTGAAAGGATGCATGGACTCTG
AACATCTAAACAGTATCGCAGACTTGCGGTCCTACAACGAGGCAAAAAGCCCCG
AAGAAAAGTGCTTCCTCGGTTGCATGCTGAAGAAGTTCAAAGCGTTGGACGCAG
ACGGGCAGTACGACGCTGAGGGCCTCAAGGCCACCATCCAGCACTGCCCCAGGA
TGAAAGCCCACCCCAACATCCAGCAGGCGGCGCTGCAGGTGGCGGACGAGTGCG
CCGGGAAAGTTACCGGATGCAGCGACTACTGCACTTGCGCACCCCTGGCCACCAG
GTGTCTCCACGAGGGAATGAAGAACAAGTCCTTCCAGACGATATTCATTGCTCTG
GATGAAGCCCTCGACAAGATGCAATCC
>LMIGOBP5 **AFL03411.1**
ATGGCGCTTGCTGCCCGAGTCTTTTCAGCTACCTTGATGTTGACTGCAGTCCTGTT
CGGCGACATCAGCACAGCAGGAGAAGTGTTCACCATGAGCCAGCTAAAGGCGGC
CGTCAACGAATGCAACGACACATACTTCTTATCCCAAAAAAACTGGGACACCGTA
TTCACCACCGGTTCTCTTGAAGACGAAAATGATCTGGTGGCGAAGTGCTTCTTCG
AGTGCGTGCTGGAGAAAACAGGGGCGATGGACGAAAAGGGGAACATCAACAGT
GACATCACGAAAGCTGTATTCCTCGCCAGCCACGAAGGAACCGGCACAGCTGTA
CAAGGCCACGATGACCTGATCGACATGTGTGTCCCAGGAAGAGATGAGACAGAC
ATCTGTGAAAGGGGATATGCACTCGTGAAGTGTGTTACAGTGGAGGAGCTGTCAC
GGCGCCAAGCACGTAAA

>LMIGOBP6 **AEX33162.1**
CAGCTCCTCCTCGTCTCCCTGGCGCTCTGCCTCTCCGCTGCCGTCGCGGAAAAGCA
GGCACCGTGGTGCCCCACGACGGCGTCCCAAGGTGTTCAGGAGGACATGGGACA
GTGCGCGGAGGAGATCAAGGACGCCATACTCAGGGAATACGCCAAGACTGTATC
AAGCAGGCGGACGAGGAGCGCAGAAATGTCAGAGGAGGACCGCCTGCTGGTTGG
GTGCATGGTGAGCTGCCTGTTCCGCAAGGGCCCGCACAGCCGTTTGCAGACGGGC
AGCAAGCTGTTGCTGGCGGAGCTTGGAGCTATGCGGCTGTTCTCTGACGGCGCCG
ACGACGCGCGGTACCGAAACGCCACGGCCACCGCCGTCAGGCGCTGCTCAGCCT
CCAGCAGGTCCCTGCTGCCCGACGACGGCGGACCCCGTCACGAGTGCGAGCTGG
GATTCTTCATGTTCGAGTGCGTCAGCGACCAGATCACTGAGTACTGCCAGTGGCA
GCCGGAG
>LMIGOBP7 **AEX33163.1**
GCGGCTGAGACCAAAGTAATGGAGGGCATAAAGGCGTGCATGGCCTCTGAGCAT
CTGGGTAGCCTGGGTCAGCTCAAAGCCAACAATGAGGCCAGGACGCCAGAGGAG
AAGTGTTTCGTCGGCTGCGTGATGAAGCACCTTCATGTGCTGAACTCTGAGGGCC
AGTACGACTTGGCTCTGGTCAAGGAGCGGGCCAACAACTGCCCTGAGCTGGCCA
AGGACCCGCAGAAGAAGGCGGACACCCTGCGGGTCGCCGAGGACTGCGCTGCCA
AAGTGATCGGGTGCAGCGGCTACTGCGAGTGTGGCGTCGCCGCCGGCGAATGTCT
GGCGCAGGGGATGGAGGCCAAGGGCCACGAGACAATATATGATTTTCTTCGAAA
AATCGTCGACAAGATGGACGTT
>LMIGOBP8 **AEX33164.1**
GCTGTACGACTGCTGCTTCTGCTGCCGCCGCTGTTGTTGCTGGCACTCAGCTGCGT
AACCGCTGCGCCCAGCATCACGTCGACAGAAATGAGGATGGATATGATGGTGAT
ACAACACTGCAACGAAACGCACCCTGTTGCGCTTATTGACATGAACAAAGCACTG
ATTAATAAAAAGATTGAGCCACAAAACACTGTCTTCAAGTGTTTCGTGTTCTGCC
TCCTCAACAAATATGAGTGGATGGACGACGAAGGCGGCTTTCTGATAGCAAACAT
GAAACACAATTTGTCCGACTCTCACCTCGACCAATTAAGCATCGATTTTATTGTTT
ACAAGTGTTCTGCGACGGGATCATCGGATAAATGTGAACGAGCATATCGATTTAC
AGAATGCTTCTGGGGAGAGGTAACCAAGTTTCCCGAGAACTCTGACGAGAAGTA
CGAAGACCCCGATTTATTTGCACTGTATCAG
>LMIGOBP9 **AEX33165.1**
ATGGACAAGGCCAGCTCTGCTGCCGTCACCGCCTGCCTGCTCATCGCGGTCGCCG
CCCTACACACGCAGGCCCTGTCGTTGGAGCAGCTAAGGCAGACAAGTAAAATAG
TGAGGAACATGTGCCTCAAGAAGACAGGTGTCGATTTAGCACTTGTCGAAGGAAT
CCAGGAAGGGCAGTTTCCTGATAACCAAGATCTAAAGTGCTACATGAAGTGCTGC
ATGGGTGCAATGCAGGTGCTGCGCCAGGGCCGGTACAACGTGAACGCCGCCAAG
AACCAGGCGGACAAGATGCTGCCGCCGGACCTCAAGGGCAGGTTCATCGACATG
CTGGACGCCTGCAGCGACCGCGGAGATGGAGTTGATGATGACTGCGAAATGGCT
TATCAGTTGACTAAATGCAGCTACGAAACGGACAAAGAGATATTCTTATTCCCT
>LMIGOBP10 **AEX33166.1**
GCGATCTCGGAGTCAATGTCTCGGGCTGAAGAAGCAGCTTCAAAGATTGATATTC
CTGAGCTGTTTGAGGAATGCAACGAGACTTTCACCATTCCTAAAGTTACTTTGAA
CTACTTTTTCTCACATGGCAGACTGCAGAACGAGAACGACTACGGATCTAAGTGC
TTCGTACACTGCCTGACGGACAGGTCCGGTGAGATTGATTCTGATGGTAACTTCG
ATGTCGACTTGATCAAAGTGATGACTCGCCGTTTCCCTAACGAGACGAACATCGA
GGGTCTCAATGAAATGGTTGAGACCTGCGTTGCTGACAGAGGCGAGACTGATTTC
TGTGAAAGAGCGTACGGTCTCGTAAGCTGTTTAGTTAAAGAGAAACTGGCGAGA
CTAGGAAACTCTCAC
>LMIGOBP11 **AEX33167.1**
ATGAGGTCGTTGCTGCCCGTCGCCGTGAGCGCCGTGCTGTTAGTAGCCCCATCAA
AGACACTCGAACCAGACTTCACAAAAGGAATCAGCGATGTAAAAGCCTGCATGG

CCTCTGAGAACCTAGATAGTCTGGATGCGCTCAGAACCAACAAGGAGGCGAGAA
CGGCAGAAGAGAAGTGCTTCATCGGCTGCATAATGAAGTTCGTTGAAGTGCTCAA
CTCCGATGGCCAGTACGACGTGGCTCTTTTCAAGGATCACATCAACGGGTGTCCT
GAAATGGCAAAGGACCAGCAGAAGAAAGCAGCTTTGTTAGAAGTTGCCGAAAGC
TGCGCTGGAAAGGCTTCGGCGTGCAGCGGCCACTGTGAATGCGGAGTCATCGTTG
CCAACTGCTTGGCCGACGAAATGGAGGCCAAGGGACAAGAGACAATATATGACC
TGCTTGAAACAATCTTCGCCAAGATGGATGCT
>LMIGOBP12 **AEX33168.1**
GCGGTCATCCTCACTGCAGCATCCACCCTCTGGTTCGCGGCAGCGGCTTTCGCTGC
CATGGTCACTACGGAGATCCCCACGGAGGATATACTCCAGCGGGTACAAGTATGT
AACAAGACCTATCCTGTTTCTCAAGAAATGCTGCGCTCCTTAGCGTCTACCGGTG
GGCTTCTTTCTGATGAATCAGACGTTAATACTCGGTGTTACCTGGAATGTTACGAG
CGCTTAGGAGGAACAGTAAACAAAGATGGAAAGTTTAACCCAGAGAAGGCTGTA
ACTCTGCTGGTAAGCTACTATCCTAAAATAGCAGAGCTTGGTGTCGACTCTGTGA
CGGAAATCCTCAAAAATTGCAATTCCAAGTCTGGGACCGGTCAGTGTATGACATC
TTATCTGATCAGAAATTGCTTCATCGCGGGTCTTAATGCTAAATCTCCGCATACAT
CTGTGTTTGATACTTCATCTTCACATATT
>LMIGOBP13 **AEX33160.1**
ATGAGCGCTCTCTTCACCTGCTGCGTGGCCGCGTGGCTGCTGCTTGCAGCGGCTTT
GCTTCAGCCGACAAAGGGTGACGACGTATGGCACAACACCGACATCCCAGCCAC
AATGGCGGAATGCAACGCTACATTCAGATTAGGATGGAGATGCTGGGATAATCT
ACTCAGTGATGGTCATGTAATTGATGAGAGTAAATACCAGCAAAAGTGTTGGTTC
TACTGCTTGCTGGATGAGACGGGATCGATGCATGCAGACGGTGCTTTCGACAAAG
ACCTGCTAAAGACGGTGCTTCAGGGCTTCCCCAACGGATCCAGTCTGGCACACCT
CGACGAGACAACCTACACCTGTGTCGCACAGAGGAATGAAGTCGATCTCTGTGA
AAGAGCATATGCCGTTGTTAAATGCATTATGACTGAGGAATTATCAAGAATGCAC
CAGTCCAGC
>LMIGOBP14 **AEX33161.1**
ATGTGTCTGGTCGTGTTATTGTTTGGCTTTGTACAAAGTCTTTCGTTGTTGTTTGTG
CCTGACTCATGTTTGTTGTTGTTTGTTGTTGTTATGTTTTATTTTCCATGTAAGATA
TCACAAGCTAATGGCTCATTTGTAATGACAATGCTGTGTCTGGAGCAAACTTGCA
AAATGATGGATCAGCTTCACCAGACATGTGTAGGAGAGAGTGGTGTATCGGAAG
GTAACATTGATGCTGCTAGGAAAGGCAATTTCATTGACGATGGCAATCTGAAGTG
CTACATGAAGTGCATATTCGTCCAAATGACATGTATGTCAGATGACGGAGTATTT
GATGCTGATACTGCAATTGCAATGTTGCCTGATAATCTCAAAGATGTTGCATCAA
AGGCACTTAATGCCTGCAAGGGTGAAAAGGGCAGCGATGCTTGTGACACAGCTTT
CAAAATAAATCAGTGCCTCTTCAAGCAGGCACCAAAGGACTACATACTTGTC
>LMIGOBP15 **KU865299.1**
ATGTTCTATTTCTACGCGTTTACGTTTTGTTGCTTGTTGTGGATGCTCTTTCACTGT
TCTGTGAACTGCGTCAACGTCGACATCGAAACTATATGGCGGGAGTGCAATGAAA
CATTCCCTGCTTCTGAAGAGGCACTCATTTCGTTCGGCAAAAACGGCACGATACC
AGACGAGAACGATTCAGTGGCGAGATGTTTCACCGACTGTTACGGAAAGAAGAC
CACTTTGCTAACATCGGACGGAAGTCTCAACTGGACAACTCTTGATTTCCTTATGA
GGAGCTACGATATGAAGCCCACCGCAAAGGAGACATTTGGAAAGTGTCAAAAGA
ACACCAGCAACGTTGAATGTATGAAAAGCTACCTTTCACTGCGTTGTGTGGCTGA
AACTGTGGAATCACTCACTGATATAAGA
>LMIGOBP16 **KU865300.1**
ATGAACTGGGGTCTGTGGTTACCTGTTACTATAGCTCTTGTTCTGCAGCTGTCAAT
AAGTGAAGCATTAAAATGTCACACTGATGAAGACACACAAAACCCAGATGAATT
TCAAGAGGTTGCTGCAATGTGTATGAAAAATACATCAGGATCTGAACTTAACAGA
AATGACAGAGAAAGTAAAAGGAATGGAAACAATTATCACAAAACTAATTTTGAA

AACACAAATGACAACTGGAACAGTGGTGGAATGGGGCAGACCTTCCCTGGATACAATAGTGAAAATGAAGGCTATGGTTTTCGTGGGAGCGGTAGATGTAATGCAAATGGTGATGGATATAATGGTAACAGAAACAACATGAATCAAAATAATATGAATGGAATGAGGCAGAGGCCACGAAATCGTAACCGTAGATCTGGCCAGCAGTCCGAAACGGCAGATGTTGATTTGGAAGATATTGAACCATGTGCAGTTCACTGCATCTTCAGGCAGATGGGAATGCTGGGAGATGATGCTATTCCAGACAGGTCTGCTGTTGCTAAAGTCATGCTGCGCGGTGTCAAGGACACAGAAGTCAAGGACTTTGTTCAGGAAGCTGTGGAAGACTGCTTTGATCAAGTCGAGTCTGATCGAAAAGGGAGCAAATGTGAATTTTCAAAGAATGTGGCATTGTGTCTTCGACAAAAAGGACGTGAGAACTGTGAAGATTGGGGTGAGCAAGATGGTGACCAACAAAGTAACCAGAATAAAAATGGAAACAATAATGGAAATTACAGCAATAACAGTAATCAGTACGGGAACAAAAAAATGGAAC

>SGREOBP1 **MF716558**

ATGTGGGCCCGCCTCTGCAACTGCGCGGCCCTCCTGCTGCTCCTGGTCGCTGCCGCCCAAGCATGGGACGTCAACATGAAACTGACTGGACGCATTATGGATGCAGCAAAGGAAGTGGACCACAAGTGCCGTGGATCTACTGGGGTGCCAAGAGAAATGCTCCATAGATATGCTGATGGGGAAACAGTAGATGACGATGATTTCAAGTGTTACTTGAAATGTATTATGATTGAGTTTAATTCTCTCTCAGAGGATGGAGTTTTTGTTTTGGAAGAAGAATTAGAAAATATTCCTCCAGAAATAAAAGAAGAAGGCCATAGGGTTGTACACAGCTGCAAACACATAAATCATGATGAAGCTTGTCAAACAGCTTACCAGATACATCAGTGCTATAAACAGAGTGACCCTGAGCTGTACAGCCTGGTAGTTCGTGCATTTGATGCAACCATT

>SGREOBP2 **MF716559**

ATGGCCAGCCATTGCCACGCCACCGTAGCCGCCGTGGTTGTCGCTGCTGCCTTTGCCGCAGTCGTCGCTGAGTCTCCGCCGCATATGGCAGCTTTCACGTTCACGAAAGAAATGTTGAGCACATGTCAAGAGAAGTCGGAGATATCACAAGAAATGGTTGACGAAATGCAGAAGAACAAAGGCTTATTACCTGATGAGAGTTCAGTTGCGCAAAGGTGTTTTCAAGAATGCATGGCTAAAGAGATGGGACTGCTTAAAAACGGTGGCGGGGTTGCGGTGGACAACATCGTGACGGTGCTGAAAGCGGCGCTTCAGATGGCTTCCGAAGGGTCCGAGGACACATACACGATCGACACGGATGCAGTGACGCGGGATCTCGAGGGCTGCCAATTCGAAGGTGAAGACGACGAATGCAACAACTCCCATGATACCATGAAATGCTTGCGGAGTTTAGGAAATCCGGAGAATATGAAGCGCTACATAACAAAGGAATCA

>SGREOBP3 **MF716560**

ATGCTGCTGGCAGCCCCCGCAAAGGCAGACGAGCCAGACCTGACAAAAGCAATCAAGGATTTAAAGGACTGCATGGCCTCTGAGAACCTAGATAGTCTGGATGGGCTCAAAACAAACAAGGAGGCCACAACAACAGAGGAGAAGTGTTTTATCGGCTGCATGATGAAGTCCGTTCATGTGCTCAACTCTGATGGTGAATACGACGTGGATTTTCTGAAGGAACACATCAATCACTGTCCTGAGCTAATGAAGGACCAGCAGAAGAAAGCAGCTGCGATCGAAGTCGCCGAAAGCTGCGCTGCGAAAGTTACAGGATGCAGTGGCTACTGCGAATGCGGGGTCGTCGCAGGCGACTGCATGTCCGAGGGAATGGAGGCCAAGGGCTACGAGACAATATATGAATGGCTTGAAAAAGTCATCGTGAAGGTGGATGCT

>SGREOBP4 **MF716561**

ATGAAGAGCTACTTCGCTTTCGTATTTGCAGCTGTCGCTCTCTTCGCCGTCGCTAAGGCAGACCCTGCGAAGCTGAAACAAGCTGTAGAAAAGTGCAAGGCATCTGAAAACCTAGACAGTTTGGATGGAATCAAGGCTAACAGACAACCCTTCACCAGTGAAGAGAAGTGCTTTCTTGGCTGTATGACACTGGATATGAAATTTCTCTCTGCAGATGGACAGTATGATGCTGCTAGCACTAAACAAATGATAAACAACTGTGAACACCTTAAGGACAAGCCTGATGAGAAGAGTGCTGCTTTGGCAGTTGCAGATGACTGTGGCAAAACAGTTACTGGATGCAATGGCTACTGTGAGTGTGGTCCCATGACTGTTGGCTGCTT

GATAAAGGGGATGATGGCTAAAGGCTATGAGGAATCATTTGCCAGAATTGACAAAGTACTTCAAAAACTTGATGGA

>SGREOBP5 **MF716562**

ATGGACAAGGGGACCACCGCGGCGCTCACCGCCTCCCTGCTGATTGTGGTCGCCGCCGTTCACACGCAGGCCCTGTCATTGGAGCAGCTGAGGCAGACAAGCAAAATAGTGAGGAACATGTGCCTCAAGAAGACATCAGTCGACCTAGCACTTGTGGAAGGAATCCAGGAAGGGAAGTTTCCTGATGACCAAAACCTAAAATGTTACATGAAATGCTGCATGGGTGCAATGCAGGTGCTGCGCCAGGGCCGTTACAACGTCAACGCTGCGAAGAACCAGGCTGAGAAGATGCTGCCCCCTGACCTCAAGGACAGATTCATAGCCATGCTGGATGCTTGCAGTGACCAAGCAGTTGGAGAAGATGATTGTGAAATGGCATATCAATTGACTAAATGCAGTTATGAAGCTGACAAAGAGATTTTCTTGTTCCCA

>SGREOBP6 **MF716563**

ATGAAGGCACACACGGCGTCGCTGGCAACGGCTACGGTGGTGATACTGCTTCTGGTAGCGGCGGTGGTCCGGGGTCAAGATGATGACATGAAGGAAATGATGGAGCAGCTTCACCAGTCATGTCTTGGTGAGAGTGGTGCATCAGATGCTAACATTGATGAAGCTAGGAAGGGGAATTTCATTGAGGATGGCAATTTGAAGTGCTACATGAAGTGCATATTCGTCCAAATGACATGTATGTCAGATGATGGAGTCTTTGATGCTGATACTGCAATTGCAATGTTGCCTGATAATCTCAAAGATGTTGCATCAAAGGCACTTACTGCCTGCAAGGATGAAAAGGGCAGTGATGCTTGTGATACAGCATTCAAAATAAATCAGTGCCTCTTCAAGCAAGCACCAAAGGACTACATACTTGTC

>SGREOBP7 **MF716564**

ATGGCGACCACCGTGCTCTCTCCGGCAGCGCTGCTGCTGGCTCTGCTGTTGGCGGCTGGTAGCGTCACCGCTCGCAGGGGTCCTAGACCTTTCGGCCGATGTGCTTCGTCGGTGGGTGATATAGATAGAGATACTATGATGAAAATCGTCAGAAACTTTGAAGTTCCCGATGAAACCTCTGATGATCAAAAGTGCATGCTCCGCTGCGCTTTAATGACAAATCGATTGGTTCGAGACGGTGTGCTGGACACGAGGCAGATCCTGATGAATATTCGAGCGGACGCGCACTTTGCGGGCAGGATGGCCTCCGTCTACGGCCAGAACATCACCCTCGACATGGACCGGCTATCCAACGACGTGGAAGCCTGTGTCACCTCTGAAGAGAGTCCTGACTGTGATACAGTCTACAACCAGTTCAAGTGCGTCTCTGATTTAATTTCCAACGGTAACATGGCAAGCTACGCCAGCTTCGTGGAGGTGGACGGGTCAGAGACGGGCGACTGGATGCGGCGGCGGCAGATGCTGGCGAGCCGCCCCCACGGTCCCCCAGGCCCCATGGGGGGCCACTGGGGGCCACCGCCCCCACACCACCGCGGAGGACCCCGCGGGGGGCGGCAGGCCTCCTCCGCCACCTCCAGAGTCCGACGAGAACGACGTCGAAGAACTGGAG

>SGREOBP8 **MF716565**

ATGAGGACCTCGGCAGCCGCCACTGGAGCCGCGCTCCTCGTGTTCGTCGCCGTCGTATCGGCAATGGAGATGACACCAGAATTCATGGAGATCATTAACAAATGCAAGGCAGAACATGAGCCCACTGAAGATGAGCTGAAAGGAATCATGATGATGAAGGTTCCAGAGAGCGAACACGGCAAGTGCTTCATGGGATGCGTTCTGCAGGAAGTTGGAGTGGTTAAGGATGGCAAGTTCGACAAGGAGGAGGCGAAGAAACACGCCGCGGCCAAGATGTCCGACAAAGACGAGCTGGAGAAGCACATGCAGCTCATTGACAAATGCAGCCAAGAAGTCGATGGCGAGACGGACTCCTGCGGAATCGGCCCGAAGCTCATGGAATGCATCAAGCAGTTTGCACCAGAGTTTGACATTGCCCTCCCACACGCCCCGTCAGAA

>SGREOBP9 **MF716566**

ATGCAGGCCCCGCAGCTCGTCTTCGTCTTGGCGCTCTGCCTCGCCTCTGCCGTCGCGGTGCTGCAGGCGCCGTGGTGCCCCACGACGGCGTCGAGCGGTGTGCAGGACGACATGGGACTCTGCGCCGAGGAACTGAAGGACGCCATCCTCAGGGAATACGCCAAGTCAGTAGCAGCCAGGCGCACGAGGAGTGCAGAAATGTCAGACGAAGACCGTCTGCTGGTTGGGTGCATGGTGAGCTGCCTCTTCCGCAAGGGACCCCACGGCCGCCTGCAGACGGGCAGCAAGCTGGCGCTGGCGGAGCTGGGCGCGATGCGGCTGTTCTCT

GACGACGCCAAAGACGCCCGGTACCTGAACGCCACGGCGGACGCTGTGAGGCGCTGCTCGGCCGCCAGCAGGTCGCTGCTGCCCGACAATGGCGGGCCACGTCATGAGTGCGAACTGGGATTCTTTATGTTCGAGTGCGTCAGCGACCAGATTACCGAGTACTGTCAGTGGCAGAAGGAG

>SGREOBP10 **MF716567**

ATGGAGTCCGCCATGAAAACTCTCCTGGTGGTTTGTGTCGCTGCATTAGGCTTTCTGGTGGCGGCCGAGATCTCAGAGTCAATGTCTCGGGCTGAGGAGGCAGCTGCGAAGATTAACCTTCCTGAGCTATTCGAGGAATGCAACGAGACTTTCCCCATTCCTAAGGTTACTCTGAACTACTTTTTCTCCCACGGCAGACTGCAAAACGAGAACGACTACGTGGCTAAGTGCTTCATACACTGCCTGACGGACAGGTCCGGAGAGATCGATTCGGAAGGTGATTTCGATGTCGACTTGATCAAAGTGATGACTCGCCGTTTCCCTAACGAGACTAACATCGAGGGCCTCAGTGAAATGGTCGACAAGTGCGTCGCTGGCAGAGGTGAGACTGATTTCTGTGAAAGAGCGTACGGTCTCGTCAGCTGTTTAGTTAAGGAGAAACTGGCGAGACTAGGACACTCTCAC

>SGREOBP11 **MF716568**

ATGAATACACTGTTCACTTGCTGCGTTGCCGTGTGGCTGCTAATTGCAGCGGCTTTGCTTCAGCCGACCAAGGGCGACGAGATGTTGCCCAATACTGACATTCCGGCCACAATGGCGGAGTGTAACGCTACTTTCAAATTAGGATGGAGATGCTGGGATAATCTTCTCAGTGATGGTCACGTGATAGATGAGAGCAAATACCAGCAAAAGTGTTGGTTCTACTGCTTACTGGATAGAACAGGAGCGATGCATGCAGACGGAGCTTTCGATAAAGATCTCCTGAAAATGGTACTTCAGGGCTTCCCCAACGGACCCAGTCTCGCACATCTCAACGAGACAACTTACACCTGTGTCGCACAGAGGAGTGAGGTCGACCTTTGTGAAAGGGCATACGCTATCGTTAAGTGCATCATGACTGAGGAATTATCAAGAATGCACCACTCCAGT

>SGREOBP12 **MF716569**

ATGAAGTGGAGTCTGTGGTTAACTGCTACTATAGCTCTTGTGCTGCAGCTGTCAATAAGTGAAGGGCTAAAATGTCATACTGATGAAGATTCACAAAACCCAGATGAATTTCAAGAGGTTGCTGCCATATGTATGAAGAATACATCAGGATCTGAGCTTAACAGAAGTGACAGAGAAAATAAAAGGAATGGAAACAATTATCACAAAAATAATTTTGGAAACACCAATGACAACTGGAGCAGTGGTGGTATGGGGCAGACCTTCCCTGGTTATAATAGTGAAAATGAAGGCTATGGTTTGCATGGCAGTGGTAGATGCACTGCCAATAATGATGGATACAACAATAACAGAAACAACATGAATCAAAATAACATGAATGGCATGAGGCAGAAGCCACGAAATCGTAATCGAAGATCTGGCCAGCAGTCTGAAGCAGCAAATGTTGATTTGGAAGATATTGAACCTTGTGCAGTTCACTGTATCTTCAGGCAGATGGGAATGCTGGGAGATGATGCTCTTCCAGATAGATCTGCTGTTGCAAAAGTCATGCTTCGTGGTGTCAAAGATACAGAAGTCAAGGACTTTGTTCAGGAAGCTGTAGAAGACTGCTTTGATCAAGTTGAGTCTGATCGAAAAGGGAGCAAATGTGATCTTTCAAAGAATGTAGCATTGTGTCTTCGGCAAAAAGGACGAGAGAACTGTGAAGATTGGGGTGAGCAAGAAGACGACCAACAAAGCAACCAGAATAAAAATGGAAACAACAATGGAAATAACAGCAATAACAGTAATAACCAGTATGGGAACAAGAAGTGGAAT

>SGREOBP13 **MF716570**

ATGGATAAATGCAGTTTTCACTTTTGTTTGACCAACACACTGATTTACTTCAGTTTAGAACTCTCTTCAGTACTACCATGGATTACAAGAGCTGAGGTAATGAAGAGGGTCAATGTATGGACAGCAAGTGATGAGCTCAGGAAAAAGCTCTTAGATGCCTTGGAAGAATGTATAATAACTGAAAATGAAGATTTAAATAGCAGTCTCTGGTCTCCCATAAAAGGCAGTCCACCTTATGGAGGCAACACATGGAATATAGGTGTAGCTACTGCAAACAGCAACAAATCTATAAATCAGTGGAGGAGCTACAATGAAATGATGGGAAACAGGACCACAAGTGTAAACAGAGACCAAAAAGTTCATATCGATGGAGTCTATTGGAAAAATGATTCCGATGATCAAGATTACAAAAACTGGAAGGAAAGTAAATGTTTCAACAGAGGAGGTAATCATCAGATGCAGCAGAGATGCAGAAGGAGTTCTGAACTACCTGGAGGAAATGCTTTATCTTCTTGTGTGGATCAATGCTTGTTTGTGAAACTCCAG

GTGGTTGATAAGAATGGCCTTCCTGTTGAAGCTCTATTTATGGAATTATTAGATAC
CAGCATACCAGAGCAACAAATGAGAAGAAAAGCCAGGAGTGAACTGCATTATTG
TTTTCAGAAAATGGCATCAGTTGCTGAAGAAGACACCTGTACATTTGGAAAGCAA
TTTGCTAGCTGCTTGGATCTGAATGTGCAGGACATTAAGAAACATCAAAGTAATT
CATCCAACATTAATAAGTTACAT
>SGREOBP14 **MF716571**
ATGATATTTTCTGTCCGATTCTTTACAGTTACTCTGCTGTTGGGTGCAGTCCTGTTC
GATGGCATCTGTAGAGCAGAAGAGACTTTCAGCAAAAACCAGCTAAAGGCGGCC
GTGAACGAGTGCAACGACACGTACTTCTTATCTCAGAAAAGCTGGGACAGCGTGT
TCACCACTGGCTCTCTTGACGACGAGAAAGATCTGGTGGCGAAGTGCTTCTTCGA
GTGCGTGCTACTGCAGACAGGCGCGATGGACGACAAGGGGACCATCAACAGTGA
CGTCACTAAAGCTGTGTTCCTCGCCAGCCACGACGGTACAGCTGTAGACGGCCAC
GGCGAACTGATCGACATGTGTGTCCCAGGAAGAGTTGAGACAGATACCTGCGAG
AAGGCGTATGCACTTGTGAAGTGTGTTACAGTGGAGGAGCTGTCACGACGCCAG
GCACGT
>OASIOBP1 **KP293567**
ATGTGGGCCCGCCTCAGCGACTGCGCCGCCCTGCTGCTGCTGCTGGCCTCCGCCG
CCCGCGCCTGGGACGTCAACATGAAACTTACTGGACGCATTATGGATGCTGCAAA
GGAAGTGGACCACACATGTCGCACATCTACTGGGGTTCCAAGAGAAATGCTCCAT
AGATATGCTGATGGTCAAACTGTAGATGATGATGATTTCAAGTGTTACCTGAAGT
GTATTATGATTGAGTTTAATTCACTCTCAGATGATGGAGTTTTCGTTTTAGAAGAA
GAATTAGAAAATGTTCCTCCAGAAATTAAGGAAGAAGGCCATAGGGTTGTACAT
AGCTGCAAACACATAAATCATGATGAAGCTTGCGAAACAGCTTACCAGATCCATC
AGTGCTATAAACAGAGTGATCCTGAGTTGTACAGCCTGGTAGTTCGTGCATTTGA
TGCAACTATTGGTGATGAC
>OASIOBP2 **KP293568**
ATGCTGCTGCTCCTGGCGGCGGCCGTTAGGGGTCAAGATGATGAGATGAGAGAA
ATGATGGATCAGCTTCACCAGACATGTGTAGGTGAGAGTGGTGTGTCAGAAGGTA
ACATTGATGCAGCTAGGAAAGGCAATTTCATTGAGGACGCCAATCTAAAGTGCTA
CATGAAGTGCATATTCGTCCAAATGACATGTATGTCAGATGATGGAGTATTTGAT
GCTGATACTGCAATTGCAATGTTGCCTGATAATCTCAAAGATGTTGCATCAAAGG
CACTTAATGCCTGCAAAGGTGAAAAGGGCAGTGATGCTTGTGACACAGCTTTCAA
AATAAATCAGTGCCTCTTCAAGCAGGCACCAAAGGACTACATACTTGTC
>OASIOBP3 **KP293569**
ATGGACAAGGCCAGCGCCGCGGCCGCCACCGCCTTCCTGCTCATCGCAGTCGCAG
CCCTACACGCGCAGGCCCTGTCGTTGGAGCAGCTAAGGCAGACAAGTAAAATAG
TGAGGAACATGTGCCTCAAGAAGACAGGAGTGGATTTAGCACTTGTGGAAGGAA
TCCAGGAAGGGCAGTTTCCTGATAACCAAGATCTCAAGTGCTACATGAAGTGCTG
CATGGGTGCAATGCAGGTGCTGCGCCAGGGCCGGTACAACGTGAACGCCGCCAA
GAACCAGGCGGAGAAGATGCTGCCGCCAGACCTCAAGGACAGGTTCCTCTCCAT
GCTGGACGCCTGCAGCGACCGCGGAGATGGAGCTGATGATGATTGCGAAATGGC
TTATCAGTTGACTAAATGCAGCTACGAAACGGACAAAGAGATTTTCTTATTCCCG
>OASIOBP4 **KP293570**
ATGAGGACCTCCGCAGCCGCAGCCGCAGCCGCCACCGGAGCAGCGCTCCTCGTG
CTCGCCGCCGTCGCATCGGCGATGGAGATGACACCAGAATTCATGGAGATCGTTA
ACAAGTGCAAGACAGAACACGAACCCACCGAAGATGAGCTGAAGGGGATGATG
GCGCTGAAAGTTCCAGAGAGCTCCAACGGCAAGTGCTTCATGGGGTGCGTTCTAC
AGGAAATTGGAGTGGTAAAGGATGGCAAGTTCGACAAGGAGGAGGCGAAGAAA
CACGCCGCTGCCAAGATGACCGACAAAGACGAACTGGAGAAGCACATGCAGCTC
ATTGAGAAATGTAGCCAAGAAGTTGGTGGCGAGACGGACTCTTGCGGAATCGGC

CCCAAACTCATGGAGTGCATCAAGCAGTTTGCACCAGAGTTTGACATTGCCCTAC
CTAAGCCGTCAGAA
>OASIOBP5 **KP293571**
ATGAGGACCTACCTGACTCTCGTATTCGCAGCCGCCGCGCTCTTCGCCGTCGCCA
AGGCTGACGCAGAGAAGGTGAAAGAAGCTGTAGAAAAATGCAAATCATCCGAAA
ACCTAGATAGCCTGGACGGCCTCAAGAGCAACAAGGCACCCTCCACCGAAGAAG
AGAAGTGCTTCATTGGCTGTATGATGATGGATATGAAATTGCTGTCTTCAGATGG
CCAGTACGATGCCGCCAGCACGAAGGAGATGATCAACAGCTGCGAATACCTCAA
GGACAAGCCAGACGAGAAGAGCGCTGCCCTGGAGGTGGCCGACGACTGCGCCGG
CAAAGCTACTGGCTGCAGCGGCCACTGTGAATGCGGACCCAAGGCGGTTGGCTG
CCTCATAAACGGCATGGTCGACAAGGGCTACGAGGAATCGTTTGCAAGAATTGA
CAAAATGTTACAAAATCTTGAA
>OASIOBP6 **KP293572**
ATGGGCGCCGCCGTAGCCGCTGCCGTACTGCTCCTAGTGGCCGTCACCAACGCTG
AGGACTCGCTGATGGAAATAGTCATCAGGGAGGTGAAAGGATGCATGGAGTCGG
AACACCTAAACAGTATCGGAGACTTGAGGTCCTACAACGATGCAAGCAGCCCCG
AACAAAAGTGCTTCCTCGGTTGCATGCTGAAGAAGTTCAAAGCGCTGGACGCAG
ACGGCCAGTACGACGCGGAGGGCCTGAAGGCGACCATCGAGCACTGCCCCAGGA
TGAAGGCGCTGCCCAACGTCCAGAAGGCGGCGCTGCAGGTGGCCGACGAGTGCG
CCGGCAAAGTTACCGGGTGCAGCGACTACTGCTCCTGCGCGCCGCTGGCCGCCAA
GTGTCTGCACGAGGGAATGAAGAACAAGTCCTTCCAGACAATATTCATTGCCCTG
GATGAAGCACTCGACAAGATGCAATCC
>OASIOBP7 **KP293573**
ATGCTGCTGGTAGCCCCATCAAAGACACACGAGCAAGACTTCACAAAAGGAATC
AGTGATGTAAAAGTCTGCATGGCCTCTGAGAACCTAGGTAGTCTGGATGGGCTCA
GAGCCAACAAAGAGGCGAGGACGGCAGAAGAGAAGTGCTTCATCGGCTGCCTAA
TGAAGTTCGTTGAAGTGCTCAACTCCGATGGCCAGTACGATGTGGCTCTTTTCAA
GGATCACATCAACAGGTCTCCTGATCTGGCAAAGATGCAGCAGAAGAAAGCAGC
CTTGTTAGAAGTCGCCGATAGCTGCGCTGGAAAGGCTTCGGCGTGCAGCGGCCAC
TGTGAATGTGGGGTCATCGTTGCTAACTGCTTGGCCGAGGGAATGGAGGCCAAGG
GAGAAGAGACAATATATGACCTGCTTGAAAAAATCTTTGCCAAGATGGACGCT
>OASIOBP8 **KP293574**
ATGCAGGCCCCGCAGCTCCTCCTCGCCGCCCTGGCGCTCTGCCTCTCCGCTGCCGT
CGCGGCGCAGCAGGCACCGTGGTGCCCCACGACGGCGTCGCAAGGCGTCCAGGA
GGACATGGGGCAGTGCGCGGAGGAGATCAAGGACGCCATACTCAGGGAATACGC
CAAGACTGTAGCAAGCAGGCGGACGAGGAGTGCAGAAATGTCAGAGGAAGACC
GTCTGTTGGTTGGGTGCATGGTGAGCTGCCTGTTCCGCAAGGGTCCGCACAGCCG
CTTGCAGACCGGCAGCAAGCTGGCGCTGGCGGAGCTGGGCGCGATGCGGCTCTTC
TCTGACGGCGCCGACGACGCGCGCTACCGAAACGCGACGGCGACCGCCGTGAGG
CGCTGCTCCGCCTCCAGCAGGTCCCTGCTGCCTGACGACGGCGGACCGCGTCATG
AGTGCGAGCTGGGATTCTTCATGTTCGAGTGCGTCAGCGACCAGATCACAGAGTA
CTGCCAGTGGCAGCCGGAG
>OASIOBP9 **KP293575**
ATGGCAGCGGCTTTGCTTCAGCCGACAAAGGGTGACGAGGTGTGGCACAATACC
GACATCCCAGCCACAATGGCCGAGTGCAACGCTACCTTCAGACTAGGGTGGAGA
TGCTGGGATAATCTACTCAGTGATGGTCATGTGATTGATGAGAGTAAATACCAAC
AAAAGTGTTGGTTCTACTGCTTGCTGGACAAGACGGGAGCGATGCATGCAGACG
GAGCTTTCGACAAAGACCTGCTAAAGACGGTACTTCAGGGTTTCCCGAACGGATC
CAGTCTGGCACACCTCGACGAGACCACCTACACCTGTGTCGCACAGAGGAATGA
AGTCGATCTCTGTGAAAGAGCATATGCCGTTGTTAAATGCATTATGACTGAGGAA
TTATCAAGAATGCACCATTCTAGC

>OASIOBP10 **KP293576**
ATGAAAGCTCTGCTCGTGGCCTGCGTCGCTGCTCTCGGCTGCCTGGCGGTGGCGG
TGGCGGCCATCTCGGAGTCAATGTCTCGGGCTGAAGAAGCAGCTGCAAAGATTG
ATCTTCCTGAGCTGTTTGAAGAATGCAACGAGACTTTCACCACTCCTAAAGCTAC
TTTGAACTACTTTTTCTCACACGGCAGACTGCAGAACGAGAACGACTACGGATCT
AAGTGCTTCATACACTGCCTGACGGACAGGTCCGGAGAGATTGATTCGGATGGTA
ACTTCGATGTCGACTTGATCAAAGTGATGACTCGCCGTTTCCCTAACGAGACGAA
CATCGAGGGCCTCAATGAAATGGTCGAGACCTGCGTCGCTGACAGAGGTGAGAC
TGACTTCTGTGAAAGAGCGTACGGCCTCGTAAGCTGTTTAATTAAAGAGAAACTG
ACGAGATTAGGACACTCTCAC
>OASIOBP11 **KP293577**
ATGTCGCTTGCTGCACGACTCTTTTCAGTTACGCTGCTCTTGGCTCCAGTGCTGTT
CAGCGACATCAGCACTGCAGGAGAAGTGTTCACCATGAGCCAGATAAAGGCGGC
CGTCAACGAATGCAATGACACCTACTTCTTATCCCAGAAAAACTGGGACAGCGTG
TTCACCACTGGCTCTCTTGAAGACGAAAAAGATCTGGTGGCGAAGTGCTTCTTCG
AGTGCGTGCTGGAGAAAACAGGCGCGATGGACGAAAAGGGGACCATCAACAGTG
ACATCACGAAAGCTGTGTTCCTCGCCAGCCACGAAGGCACCGGCACACCTGTACA
AGGCCATGACGAGCTGATCGACATGTGTGTCCCAGGAAGAGATGAGACAGACAT
CTGTGAAAAGGGATATGCACTCGTGAAGTGTGTTACATTGGAGGAGCTGTCACGA
CGCCATGCACGTAAA
>OASIOBP12 **KP293578**
ATGTTCTATTTCTACGCGTTTACGCTTTGTTGCTTGTTGTGGGTGCTCTTTCACTGT
TCTGTGAACTGCGTCGACATCGACATCGAAACTATATGGCGGGAGTGCAATGAAA
CGTTCCCTGCTTCTGAAGAATCACTCATTTCGTTCGGTAAAAACGGCACGATACC
AGACGAGAACGATTCAACGGCGAGATGTTTCGCCGACTGTTACGGAAAGAAGAC
CACTATGCTAACATCGGATGGAAGCCTCAACTGGACAACCCTAGATTTCATTATG
AGGAGCTACAATATGAAGCCCACCGCAACGGAGACATTTGGAAAGTGTCAAAAG
GACACCAGCAACGTTGAATGTATGAAAAGCTACCTTTCACTTCGTTGTGTGGCTG
AAACAATTGCATCACTCAGTAATATAAGA
>OASIOBP13 **KP293579**
ATGGTCAACCACCACCAGGGAGTCGTCGCCATCGCTGCCGCCCTCACTGCGATGG
CCGCAGCAGCGCCGTCGTCCATTGCGGAAGCTACCAGGTTCAGCAAAGAAACAG
TGAGCAAATGCCAAGAGAAATGGCAGGTATCTGAAGAAATTATCGAAGAAATGC
AAAGGAATAAAGGAGCTTTACCTAATGAGGACTCCGTTGAGCAAAGGTGTTTCGC
AGAGTGCGTGGCTAAGGAGATGGGTATGATTAATAACGGCGGCGGGGTTGCTGC
CGACAAGATTGTCAAAATGCTGGAAGCGGTGTTCCAGATGGCTTCCAAAGAAAC
AGGGGAAAAACTGAAGCTCGACTCGCGAGCATTGAAGAGGGATCTCGAGGCCTG
CCAATTCAAAGGTGAAGACGACGAGTGCACCAACTCCTATGATACGTTGAAATGC
TTGCGAACATTAGGAACTTCGGATAATATGAGGCGGTACGTAACGAAGGAATCA
>OASIOBP14 **KP293580**
ATGTTCTATTTCTACGCGTTTACGCTTTGTTGCTTGTTGTGGGTGCTCTTTCACTGT
TCTGTGAACTGCGTCGACATCGACATCGAAACTATATGGCGGGAGTGCAATGAAA
CGTTCCCTGCTTCTGAAGAATCACTCATTTCGTTCGGTAAAAACGGCACGATACC
AGACGAGAACGATTCAACGGCGAGATGTTTCGCCGACTGTTACGGAAAGAAGAC
CACTATGCTAACATCGGATGGAAGCCTCAACTGGACAACCCTAGATTTCATTATG
AGGAGCTACAATATGAAGCCCACCGCAACGGAGACATTTGGAAAGTGTCAAAAG
GACATACCAACATCTCATCAATTGAAGACACTGTGCACATCACTGAGTCTGAAGC
ACGTGAACCCTTCCCACCAATTTCATAGAGGCAGAGGACATGGTTGGCTGTTGAA
GCATGTGAACAGAGGTCCATGCACTCAGCGCTGCAAATACACG
>OASIOBP15 **KP293581**

ATGAGAACGTCACATGTTTATACGATCTTCTGCGCTATTATAGTCACATGTTACTG
TGATTCTGTCGAGGTGTCAGATGGACCGGAAGAAGCCACCATGATGAAATGTGC
AGTAGAATTAGGCTTTGGACATGATGAGATCCAGAGAATTAAGAGTTCTCCAATT
CCTGATGAAACGAATGAAAACGAAAGATGTCTGATGAAGTGCATTGGACGAAAA
ATGAAATACCTTACATCAGAAGATATCGTGGACGTCCATCATCTTCTGGAATTAT
CAGGAGAAATGATAGAAAAGGAAGGCTATACTAAAAGCGAAATGAGACAAATG
TTGGTGGAGTGCACTAAGAAAACCGGACCGAAAAATGTATGACAGCATTTAAG
AACTTGAGGTGTCTGATGAACGCATTTAAA
>CKIAOBP1 **KP255951.1**
ATGTGGGCACGCCTCTACAACTGCGCGGCCCTGCTGCTGCTCCTGGTTGCTGCCA
CACACGGCTGGGACGTCAACATGAAACTGACTGGGCGCATTATGGATGCAGCAA
AGGAAGTGGACACCAAGTGCCGTTCATCTACTGGGGTTCCAAGAGAAATGCTTCA
TAGATACGCTGATGGTCAAACAGTAGACGATGCAGATTTCAAGTGTTACTTGAAG
TGTATTATGATTGAGTTTAATTCACTCTCAGATGAAGGAGTTTTTGTTTTAGAAGA
AGAATTAGAAAATGTTCCTCCAGAAATAAAGGAAGAAGGCCATAGGATTGTACA
CAGCTGCAAACACATAAATCATGATGAAGCTTGTGAGACAGCTTACCAGATCCAT
CAGTGCTACAAACAGAGTGATCCTGATTTGTACAGCCTGGTAGTTCGTGCATTTG
ATGCAACCATTGATGCA
>CKIAOBP2 **KP255952.1**
ATGAGGACCTCAGCAGCCGCCGCCGGAGCAGCGCTCCTAGTACTCGCCGCCGTCG
CATCGGCGATGGAGATGACACCAGAATTCATGGAGATCGTTAACAAATGCAAGA
CAGAACATGAACCCACCGACGATGAGCTGAAAGGGATGATGATGTTGAAAGTCC
CAGAGAGCGAGAAGGGCAAGTGCTTCATGGGGTGCGTTCTACAGGAAATTGGAG
TGGTAAAGGAAGGCAAGTTCGACAAGGAGGAGGCGAAGAAACACGCTGAATCC
AAGATGACCGACAAAGACGAGCTGGAAAAGCACATGCAGCTAATTGAGAAATGT
AGCCAAGAAGTTGGTGGCGAGACGGACTCCTGCGGAATCGGCCCCAAACTGATG
GAGTGCATCAAGCAGTTTGCCCCAGAGTTTGACATCGCTCTCCCGCAGCCCCCGT
CAGAG
>CKIAOBP3 **KP255953.1**
ATGAGGACCTACCTGGCTCTCCTAGTTGCAGCAGTCGCTCTCTTCGCCGTTGCTCA
GGCTGGCCCAGAAGATAAGCTGAAAGAATCTGTAGAAAAATGCAAGGCATCTGA
AAACCTAGATAACTTGGATGGTCTCAAGACCGGCAAGGCACCCTCTACCAAAGA
AGAAAAGTGCTTCATTGGCTGTTTTGCAATGGATATGAATGTGCTGAATTCAGAT
GGACACTATGATGCTGCAAGCACCAAGGAAATGATAAACAACTGTGAACACCTT
AAGAACAAACCTGATGAAAAGAGTGCAGCCTTGGAGGTGGCTGATGACTGTGGC
CACAAAGTTACTGACTGCAGTGGCCACTGTGAGTGTGGACCCAAGGCAGTTGGCT
GCCTAATAAAGGGTATGATGGATAAAGGCTTTGAGGAGTCTTTCGCTAGCCTGGA
CAAAGTATTACAAAAAGTTGATGGA
>CKIAOBP4 **KP255954.1**
ATGCGCGTCGAGGTAGTCGCTGCCGTTCTGTTGCTGGCCGCCGTGACCAACGCCG
AAGATTCTCTGGTTGACATAGTCATCAGGGAGGTTAAAGGATGCATGGATTCGGA
ACATCTAGGCAGTATCGGGGGATTGCGATCGACCAACGACCCAAACAGCGCTGA
ACAAAAGTGCTTCCTTGGTTGTATGCTGAAGAAGTTCAAAGCGCTTGACGCAGGC
GGGCACTACGACGCAGAGGGCCTGAAGACGACCATCCAGCACTGTCCCAGGATG
AAGGCCCACCCCGACATACAGAAAGCTGCAATGCAAGTGGCCGACGAGTGCAAC
GGCAAAGTTACTGGCTGCGACGACTACTGCTCGTGTGCGCCTTTGGCCAGCAAGT
GCCTGCATGAAGGAATGAAGAACAAAGCCTTCCAGACCATATTCATCGCTCTGGA
TGAAGCACTTGACAAGATGGAGTCC
>CKIAOBP6 **KP255956.1**
ATGAAAGCTCTGCTGGTGGCGTTCGTCGCTGCACTGGGCTGCCTGGCCCTGGCTG
TGGCCGCCATCTCGGAGTCGATGGCTCGGGCTGAAGAAGCAGCTGCGAAAATCG

AACTTTCGGAGCTATTTGAGGAATGCAACGAGACATTCCCCATTCCTAAAGCTAC
TATCAACTACTTTTTCTCACATGGTAGACTGCAAAACGAGAATGACTACGGATCT
AAGTGCTACATACACTGCCTGACGGACAGGTCCGGGGAGATTGATTCGGATGGTA
ACTTCGACGCCGACATGATCAAAGTGATGACTCGCCGTTTCCCCAACGAGACTCA
CATCGAGGGCCTCAACGAAATGGTTGACGGCTGCGTCGCTGCTAGAGGTGAGAG
TGATTTCTGTGAAAGAGCGTACGGTCTCGTCAGCTGTTTAATCAAAGAGAAACTG
GCGAGACTTGGACACTCTCAC
>CKIAOBP7 **KP255957.1**
ATGAGGACAATCCTACTTGTCTCCGTCAGCGCGATGCTGCTGGCCGCCACGGAGG
CAGACGACGCAGACCAGATGAAAGTAATCAGCGAAATAAAAGCGTGCATGGCAT
CTGAGAACCTAGATAGTCTGGACCCGATCAGAACCAACAACGAGGCCAGGACAT
CACAGGAGAAGTGTTTTATCGGCTGTATGATGAAGAACCTTCATGTGCTCAACTC
TGACGGCCAGTACGACTCGGCTCTCCTCAAGGAGCACATGAGCCACTGCCCTGAG
ATGGCCAAGGACCCGCAGAAGAAGGCAGATACCCTCCTAGTCGCCGAAGATTGC
GCCGCCAAAGTTACCGGGTGCAACGGCTACTGTGAGTGCGGCGTCGTGGCTGGCA
ACTGTCTGGCACAGGGAATGGAGGCCAAGGGCCATGAGACAAAATATACGACTG
GCTTCGAAACATCGTGGAGAAGATGCATGCTTAGATCAGGACCAAGTGGAACT
>CKIAOBP8 **KP255958.1**
ATGGCGGTCTCCGTTATTGCCGGTGCCCTTGGGCTCCTAGCAGCGGCTCTGGCTAC
TACAGTCACAACTGACATCCCCACGGAGGAGATTCTGCGTTGGGTAGAAACATGT
AACAAGAGCCACCCCATTTCTCAAGAACTGCTGCGATCCTTGGCGACTAGCGGTG
GTCTTCTAGCTGATGAATCAGACACTAATGCCCGATGCTACCTGGAGTGTTACGA
CCGTCTAGTGGGAGTAGCAAACAGCGATGGAATGTTGAATGTGGAGAATGTTGT
AGCTATTTTGATACACTACTATCCAAAAATAGCAGAGATTGGAGCCGAATCTGTG
GCGGAAATCGTCAGAAATTGCAGTTCTAAGTCTGGGACCGGTCAGTGTATGACAT
CATACTTGATCAGAAAGTGCTACACCGAGGGTCTAGGGGTGAAGTCACCAAATGT
GTCGATATTTGATTCTTCTTTTTCA

>LMIGOBP1
MWARLNDCAALLLLLAAAARAWDVNMKLTGRIMDAAKEVDHTCRSSTGVPRDML
HRYAEGQTVDDDDFKCYLKCIMVEFNSLSDDGVFVLEEELENVPPEIKEEGHRVVHS
CKHINHDEACETAYQIHQCYKQSDPELYSLVVRAFDATIGD
>LMIGOBP2
WIPGLRNSNSDMRTSAAAAATGPAFLVLAAVASAMEMTPEFMEIVNKCKTEHEPTE
DELKGMMALKVPESANGKCFMGCVLQEIGVVKDGKFDKEEAKKHAASKMTDKDE
LEKHMQLIEKCSQEVGGETDSRGIGPKLMECIKQFAPEFDIALPQQPSE
>LMIGOBP3
DAEKMKEAVDKCKASENLDSLDGLKSSKSPSTEEEKCFIGCLMMDMKLLSSDGQYD
AASTKDMINNCEYLKDKPDEKSVALEVADDCAGKATGCSGHCECGPKAVGCLIKG
MVDKGYEESFARIDKMLEKLDD
>LMIGOBP4
MGTAVAAAVLLLVAVANAEDSLMEIVIREVKGCMDSEHLNSIADLRSYNEAKSPEEK
CFLGCMLKKFKALDADGQYDAEGLKATIQHCPRMKAHPNIQQAALQVADECAGKV
TGCSDYCTCAPLATRCLHEGMKNKSFQTIFIALDEALDKMQS
>LMIGOBP5
MALAARVFSATLMLTAVLFGDISTAGEVFTMSQLKAAVNECNDTYFLSQKNWDTVF
TTGSLEDENDLVAKCFFECVLEKTGAMDEKGNINSDITKAVFLASHEGTGTAVQGHD
DLIDMCVPGRDETDICERGYALVKCVTVEELSRRQARK
>LMIGOBP6

QLLLVSLALCLSAAVAEKQAPWCPTTASQGVQEDMGQCAEEIKDAILREYAKTVSSR
RTRSAEMSEEDRLLVGCMVSCLFRKGPHSRLQTGSKLLLAELGAMRLFSDGADDAR
YRNATATAVRRCSASSRSLLPDDGGPRHECELGFFMFECVSDQITEYCQWQPE
>LMIGOBP7
AAETKVMEGIKACMASEHLGSLGQLKANNEARTPEEKCFVGCVMKHLHVLNSEGQ
YDLALVKERANNCPELAKDPQKKADTLRVAEDCAAKVIGCSGYCECGVAAGECLA
QGMEAKGHETIYDFLRKIVDKMDV
>LMIGOBP8
AVRLLLLLPPLLLLALSCVTAAPSITSTEMRMDMMVIQHCNETHPVALIDMNKALINK
KIEPQNTVFKCFVFCLLNKYEWMDDEGGFLIANMKHNLSDSHLDQLSIDFIVYKCSA
TGSSDKCERAYRFTECFWGEVTKFPENSDEKYEDPDLFALYQ
>LMIGOBP9
MDKASSAAVTACLLIAVAALHTQALSLEQLRQTSKIVRNMCLKKTGVDLALVEGIQE
GQFPDNQDLKCYMKCCMGAMQVLRQGRYNVNAAKNQADKMLPPDLKGRFIDMLD
ACSDRGDGVDDDCEMAYQLTKCSYETDKEIFLFP
>LMIGOBP10
AISESMSRAEEAASKIDIPELFEECNETFTIPKVTLNYFFSHGRLQNENDYGSKCFVHC
LTDRSGEIDSDGNFDVDLIKVMTRRFPNETNIEGLNEMVETCVADRGETDFCERAYG
LVSCLVKEKLARLGNSH
>LMIGOBP11
MRSLLPVAVSAVLLVAPSKTLEPDFTKGISDVKACMASENLDSLDALRTNKEARTAE
EKCFIGCIMKFVEVLNSDGQYDVALFKDHINGCPEMAKDQQKKAALLEVAESCAGK
ASACSGHCECGVIVANCLADEMEAKGQETIYDLLETIFAKMDA
>LMIGOBP12
AVILTAASTLWFAAAAFAAMVTTEIPTEDILQRVQVCNKTYPVSQEMLRSLASTGGL
LSDESDVNTRCYLECYERLGGTVNKDGKFNPEKAVTLLVSYYPKIAELGVDSVTEIL
KNCNSKSGTGQCMTSYLIRNCFIAGLNAKSPHTSVFDTSSSHI
>LMIGOBP13
MSALFTCCVAAWLLLAAALLQPTKGDDVWHNTDIPATMAECNATFRLGWRCWDN
LLSDGHVIDESKYQQKCWFYCLLDETGSMHADGAFDKDLLKTVLQGFPNGSSLAHL
DETTYTCVAQRNEVDLCERAYAVVKCIMTEELSRMHQSS
>LMIGOBP14
MCLVVLLFGFVQSLSLLFVPDSCLLLFVVVMFYFPCKISQANGSFVMTMLCLEQTCK
MMDQLHQTCVGESGVSEGNIDAARKGNFIDDGNLKCYMKCIFVQMTCMSDDGVFD
ADTAIAMLPDNLKDVASKALNACKGEKGSDACDTAFKINQCLFKQAPKDYILV
>LMIGOBP15
MFYFYAFTFCCLLWMLFHCSVNCVNVDIETIWRECNETFPASEEALISFGKNGTIPDE
NDSVARCFTDCYGKKTTLLTSDGSLNWTTLDFLMRSYDMKPTAKETFGKCQKNTSN
VECMKSYLSLRCVAETVESLTDIR
>LMIGOBP16
MNWGLWLPVTIALVLQLSISEALKCHTDEDTQNPDEFQEVAAMCMKNTSGSELNRN
DRESKRNGNNYHKTNFENTNDNWNSGGMGQTFPGYNSENEGYGFRGSGRCNANGD
GYNGNRNNMNQNNMNGMRQRPRNRNRRSGQQSETADVDLEDIEPCAVHCIFRQMG
MLGDDAIPDRSAVAKVMLRGVKDTEVKDFVQEAVEDCFDQVESDRKGSKCEFSKN
VALCLRQKGRENCEDWGEQDGDQQSNQNKNGNNNGNYSNNSNQYGNKKWN
>SGREOBP1
MWARLCNCAALLLLLVAAAQAWDVNMKLTGRIMDAAKEVDHKCRGSTGVPREML
HRYADGETVDDDDFKCYLKCIMIEFNSLSEDGVFVLEEELENIPPEIKEEGHRVVHSC
KHINHDEACQTAYQIHQCYKQSDPELYSLVVRAFDATI
>SGREOBP2

MASHCHATVAAVVVAAAFAAVVAESPPHMAAFTFTKEMLSTCQEKSEISQEMVDEMQKNKGLLPDESSVAQRCFQECMAKEMGLLKNGGGVAVDNIVTVLKAALQMASEGSEDTYTIDTDAVTRDLEGCQFEGEDDECNNSHDTMKCLRSLGNPENMKRYITKES
>SGREOBP3
MLLAAPAKADEPDLTKAIKDLKDCMASENLDSLDGLKTNKEATTTEEKCFIGCMMKSVHVLNSDGEYDVDFLKEHINHCPELMKDQQKKAAAIEVAESCAAKVTGCSGYCECGVVAGDCMSEGMEAKGYETIYEWLEKVIVKVDA
>SGREOBP4
MKSYFAFVFAAVALFAVAKADPAKLKQAVEKCKASENLDSLDGIKANRQPFTSEEKCFLGCMTLDMKFLSADGQYDAASTKQMINNCEHLKDKPDEKSAALAVADDCGKTVTGCNGYCECGPMTVGCLIKGMMAKGYEESFARIDKVLQKLDG
>SGREOBP5
MDKGTTAALTASLLIVVAAVHTQALSLEQLRQTSKIVRNMCLKKTSVDLALVEGIQEGKFPDDQNLKCYMKCCMGAMQVLRQGRYNVNAAKNQAEKMLPPDLKDRFIAMLDACSDQAVGEDDCEMAYQLTKCSYEADKEIFLFP
>SGREOBP6
MKAHTASLATATVVILLLVAAVVRGQDDDMKEMMEQLHQSCLGESGASDANIDEARKGNFIEDGNLKCYMKCIFVQMTCMSDDGVFDADTAIAMLPDNLKDVASKALTACKDEKGSDACDTAFKINQCLFKQAPKDYILV
>SGREOBP7
MATTVLSPAALLLALLLAAGSVTARRGPRPFGRCASSVGDIDRDTMMKIVRNFEVPDETSDDQKCMLRCALMTNRLVRDGVLDTRQILMNIRADAHFAGRMASVYGQNITLDMDRLSNDVEACVTSEESPDCDTVYNQFKCVSDLISNGNMASYASFVEVDGSETGDWMRRRQMLASRPHGPPGPMGGHWGPPPPHHRGGPRGGGRPPPPPPESDENDVEELE
>SGREOBP8
MRTSAAATGAALLVFVAVVSAMEMTPEFMEIINKCKAEHEPTEDELKGIMMMKVPESEHGKCFMGCVLQEVGVVKDGKFDKEEAKKHAAAKMSDKDELEKHMQLIDKCSQEVDGETDSCGIGPKLMECIKQFAPEFDIALPHAPSE
>SGREOBP9
MQAPQLVFVLALCLASAVAVLQAPWCPTTASSGVQDDMGLCAEELKDAILREYAKSVAARRTRSAEMSDEDRLLVGCMVSCLFRKGPHGRLQTGSKLALAELGAMRLFSDDAKDARYLNATADAVRRCSAASRSLLPDNGGPRHECELGFFMFECVSDQITEYCQWQKE
>SGREOBP10
MESAMKTLLVVCVAALGFLVAAEISESMSRAEEAAAKINLPELFEECNETFPIPKVTLNYFFSHGRLQNENDYVAKCFIHCLTDRSGEIDSEGDFDVDLIKVMTRRFPNETNIEGLSEMVDKCVAGRGETDFCERAYGLVSCLVKEKLARLGHSH
>SGREOBP11
MNTLFTCCVAVWLLIAAALLQPTKGDEMLPNTDIPATMAECNATFKLGWRCWDNLLSDGHVIDESKYQQKCWFYCLLDRTGAMHADGAFDKDLLKMVLQGFPNGPSLAHLNETTYTCVAQRSEVDLCERAYAIVKCIMTEELSRMHHSS
>SGREOBP12
MKWSLWLTATIALVLQLSISEGLKCHTDEDSQNPDEFQEVAAICMKNTSGSELNRSDRENKRNGNNYHKNNFGNTNDNWSSGGMGQTFPGYNSENEGYGLHGSGRCTANNDGYNNNRNNMNQNNMNGMRQKPRNRNRRSGQQSEAANVDLEDIEPCAVHCIFRQMGMLGDDALPDRSAVAKVMLRGVKDTEVKDFVQEAVEDCFDQVESDRKGSKCDLSKNVALCLRQKGRENCEDWGEQEDDQQSNQNKNGNNNGNNSNNSNNQYGNKKWN
>SGREOBP13
MDKCSFHFCLTNTLIYFSLELSSVLPWITRAEVMKRVNVWTASDELRKKLLDALEECIITENEDLNSSLWSPIKGSPPYGGNTWNIGVATANSNKSINQWRSYNEMMGNRTTSVNRDQKVHIDGVYWKNDSDDQDYKNWKESKCFNRGGNHQMQQRCRRSSELPGGNAL

SSCVDQCLFVKLQVVDKNGLPVEALFMELLDTSIPEQQMRRKARSELHYCFQKMASVAEEDTCTFGKQFASCLDLNVQDIKKHQSNSSNINKLH

>SGREOBP14

MIFSVRFFTVTLLLGAVLFDGICRAEETFSKNQLKAAVNECNDTYFLSQKSWDSVFTTGSLDDEKDLVAKCFFECVLLQTGAMDDKGTINSDVTKAVFLASHDGTAVDGHGELIDMCVPGRVETDTCEKAYALVKCVTVEELSRRQAR

>OASIOBP1

MWARLSDCAALLLLLASAARAWDVNMKLTGRIMDAAKEVDHTCRTSTGVPREMLHRYADGQTVDDDDFKCYLKCIMIEFNSLSDDGVFVLEEELENVPPEIKEEGHRVVHSCKHINHDEACETAYQIHQCYKQSDPELYSLVVRAFDATIGDD

>OASIOBP2

MLLLLAAAVRGQDDEMREMMDQLHQTCVGESGVSEGNIDAARKGNFIEDANLKCYMKCIFVQMTCMSDDGVFDADTAIAMLPDNLKDVASKALNACKGEKGSDACDTAFKINQCLFKQAPKDYILV

>OASIOBP3

MDKASAAAATAFLLIAVAALHAQALSLEQLRQTSKIVRNMCLKKTGVDLALVEGIQEGQFPDNQDLKCYMKCCMGAMQVLRQGRYNVNAAKNQAEKMLPPDLKDRFLSMLDACSDRGDGADDDCEMAYQLTKCSYETDKEIFLFP

>OASIOBP4

MRTSAAAAAAATGAALLVLAAVASAMEMTPEFMEIVNKCKTEHEPTEDELKGMMALKVPESSNGKCFMGCVLQEIGVVKDGKFDKEEAKKHAAAKMTDKDELEKHMQLIEKCSQEVGGETDSCGIGPKLMECIKQFAPEFDIALPKPSE

>OASIOBP5

MRTYLTLVFAAAALFAVAKADAEKVKEAVEKCKSSENLDSLDGLKSNKAPSTEEEKCFIGCMMMDMKLLSSDGQYDAASTKEMINSCEYLKDKPDEKSAALEVADDCAGKATGCSGHCECGPKAVGCLINGMVDKGYEESFARIDKMLQNLE

>OASIOBP6

MGAAVAAAVLLLVAVTNAEDSLMEIVIREVKGCMESEHLNSIGDLRSYNDASSPEQKCFLGCMLKKFKALDADGQYDAEGLKATIEHCPRMKALPNVQKAALQVADECAGKVTGCSDYCSCAPLAAKCLHEGMKNKSFQTIFIALDEALDKMQS

>OASIOBP7

MLLVAPSKTHEQDFTKGISDVKVCMASENLGSLDGLRANKEARTAEEKCFIGCLMKFVEVLNSDGQYDVALFKDHINRSPDLAKMQQKKAALLEVADSCAGKASACSGHCECGVIVANCLAEGMEAKGEETIYDLLEKIFAKMDA

>OASIOBP8

MQAPQLLLAALALCLSAAVAAQQAPWCPTTASQGVQEDMGQCAEEIKDAILREYAKTVASRRTRSAEMSEEDRLLVGCMVSCLFRKGPHSRLQTGSKLALAELGAMRLFSDGADDARYRNATATAVRRCSASSRSLLPDDGGPRHECELGFFMFECVSDQITEYCQWQPE

>OASIOBP9

MAAALLQPTKGDEVWHNTDIPATMAECNATFRLGWRCWDNLLSDGHVIDESKYQQKCWFYCLLDKTGAMHADGAFDKDLLKTVLQGFPNGSSLAHLDETTYTCVAQRNEVDLCERAYAVVKCIMTEELSRMHHSS

>OASIOBP10

MKALLVACVAALGCLAVAVAAISESMSRAEEAAAKIDLPELFEECNETFTTPKATLNYFFSHGRLQNENDYGSKCFIHCLTDRSGEIDSDGNFDVDLIKVMTRRFPNETNIEGLNEMVETCVADRGETDFCERAYGLVSCLIKEKLTRLGHSH

>OASIOBP11

MSLAARLFSVTLLLAPVLFSDISTAGEVFTMSQIKAAVNECNDTYFLSQKNWDSVFTTGSLEDEKDLVAKCFFECVLEKTGAMDEKGTINSDITKAVFLASHEGTGTPVQGHDELIDMCVPGRDETDICEKGYALVKCVTLEELSRRHARK

>OASIOBP12
MFYFYAFTLCCLLWVLFHCSVNCVDIDIETIWRECNETFPASEESLISFGKNGTIPDEN
DSTARCFADCYGKKTTMLTSDGSLNWTTLDFIMRSYNMKPTATETFGKCQKDTSNV
ECMKSYLSLRCVAETIASLSNIR
>OASIOBP13
MVNHHQGVVAIAAALTAMAAAAPSSIAEATRFSKETVSKCQEKWQVSEEIIEEMQRN
KGALPNEDSVEQRCFAECVAKEMGMINNGGGVAADKIVKMLEAVFQMASKETGEK
LKLDSRALKRDLEACQFKGEDDECTNSYDTLKCLRTLGTSDNMRRYVTKES
>OASIOBP14
MFYFYAFTLCCLLWVLFHCSVNCVDIDIETIWRECNETFPASEESLISFGKNGTIPDEN
DSTARCFADCYGKKTTMLTSDGSLNWTTLDFIMRSYNMKPTATETFGKCQKDIPTSH
QLKTLCTSLSLKHVNPSHQFHRGRGHGWLLKHVNRGPCTQRCKYT
>OASIOBP15
MRTSHVYTIFCAIIVTCYCDSVEVSDGPEEATMMKCAVELGFGHDEIQRIKSSPIPDET
NENERCLMKCIGRKMKYLTSEDIVDVHHLLELSGEMIEKEGYTKSEMRQMLVECTK
KTGTEKCMTAFKNLRCLMNAFK
>CKIAOBP1
MWARLYNCAALLLLLVAATHGWDVNMKLTGRIMDAAKEVDTKCRSSTGVPREML
HRYADGQTVDDADFKCYLKCIMIEFNSLSDEGVFVLEEELENVPPEIKEEGHRIVHSC
KHINHDEACETAYQIHQCYKQSDPDLYSLVVRAFDATIDA
>CKIAOBP2
MRTSAAAAGAALLVLAAVASAMEMTPEFMEIVNKCKTEHEPTDDELKGMMMLKVP
ESEKGKCFMGCVLQEIGVVKEGKFDKEEAKKHAESKMTDKDELEKHMQLIEKCSQE
VGGETDSCGIGPKLMECIKQFAPEFDIALPQPPSE
>CKIAOBP3
MRTYLALLVAAVALFAVAQAGPEDKLKESVEKCKASENLDNLDGLKTGKAPSTKEE
KCFIGCFAMDMNVLNSDGHYDAASTKEMINNCEHLKNKPDEKSAALEVADDCGHK
VTDCSGHCECGPKAVGCLIKGMMDKGFEESFASLDKVLQKVDG
>CKIAOBP4
MRVEVVAAVLLLAAVTNAEDSLVDIVIREVKGCMDSEHLGSIGGLRSTNDPNSAEQK
CFLGCMLKKFKALDAGGHYDAEGLKTTIQHCPRMKAHPDIQKAAMQVADECNGKV
TGCDDYCSCAPLASKCLHEGMKNKAFQTIFIALDEALDKMES
>CKIAOBP6
MKALLVAFVAALGCLALAVAAISESMARAEEAAAKIELSELFEECNETFPIPKATINY
FFSHGRLQNENDYGSKCYIHCLTDRSGEIDSDGNFDADMIKVMTRRFPNETHIEGLNE
MVDGCVAARGESDFCERAYGLVSCLIKEKLARLGHSH
>CKIAOBP7
MRTILLVSVSAMLLAATEADDADQMKVISEIKACMASENLDSLDPIRTNNEARTSQE
KCFIGCMMKNLHVLNSDGQYDSALLKEHMSHCPEMAKDPQKKADTLLVAEDCAAK
VTGCNGYCECGVVAGNCLAQGMEAKGHETKYTTGFETSWRRCMLRSGPSGT
>CKIAOBP8
MAVSVIAGALGLLAAALATTVTTDIPTEEILRWVETCNKSHPISQELLRSLATSGGLLA
DESDTNARCYLECYDRLVGVANSDGMLNVENVVAILIHYYPKIAEIGAESVAEIVRN
CSSKSGTGQCMTSYLIRKCYTEGLGVKSPNVSIFDSSFS
